# DNA with zwitterionic and negatively charged phosphate modifications: Formation of DNA triplexes, duplexes and cell uptake studies

**DOI:** 10.3762/bjoc.17.65

**Published:** 2021-03-29

**Authors:** Yongdong Su, Maitsetseg Bayarjargal, Tracy K Hale, Vyacheslav V Filichev

**Affiliations:** 1 School of Fundamental Sciences, Massey University, Private Bag 11-222, 4442 Palmerston North, New Zealand; 2 Maurice Wilkins Centre for Molecular Biodiscovery, Auckland 1142, New Zealand

**Keywords:** cell uptake, charge neutral modification, DNA, modified phosphates, Staudinger reaction

## Abstract

Two phosphate modifications were introduced into the DNA backbone using the Staudinger reaction between the 3’,5’-dinucleoside β-cyanoethyl phosphite triester formed during DNA synthesis and sulfonyl azides, 4-(azidosulfonyl)-*N,N,N*-trimethylbutan-1-aminium iodide (N+ azide) or *p*-toluenesulfonyl (tosyl or Ts) azide, to provide either a zwitterionic phosphoramidate with N+ modification or a negatively charged phosphoramidate for Ts modification in the DNA sequence. The incorporation of these N+ and Ts modifications led to the formation of thermally stable parallel DNA triplexes, regardless of the number of modifications incorporated into the oligodeoxynucleotides (ONs). For both N+ and Ts-modified ONs, the antiparallel duplexes formed with complementary RNA were more stable than those formed with complementary DNA (except for ONs with modification in the middle of the sequence). Additionally, the incorporation of N+ modifications led to the formation of duplexes with a thermal stability that was less dependent on the ionic strength than native DNA duplexes. The thermodynamic analysis of the melting curves revealed that it is the reduction in unfavourable entropy, despite the decrease in favourable enthalpy, which is responsible for the stabilisation of duplexes with N+ modification. N+ONs also demonstrated greater resistance to nuclease digestion by snake venom phosphodiesterase I than the corresponding Ts-ONs. Cell uptake studies showed that Ts-ONs can enter the nucleus of mouse fibroblast NIH3T3 cells without any transfection reagent, whereas, N+ONs remain concentrated in vesicles within the cytoplasm. These results indicate that both N+ and Ts-modified ONs are promising for various in vivo applications.

## Introduction

**Figure 1:**
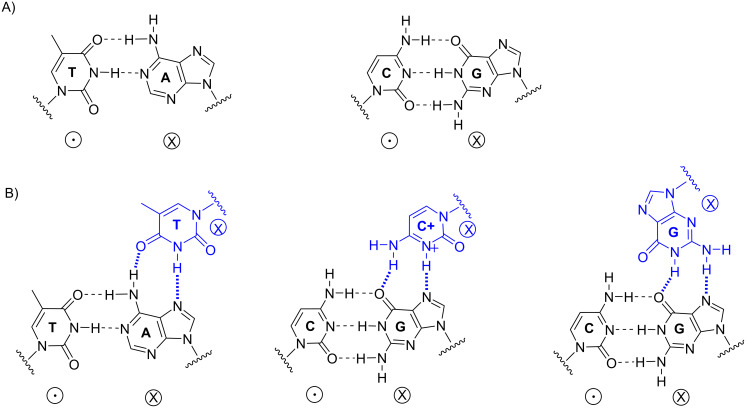
Illustration of H-bonding in a DNA duplex and a parallel triplex. A) Depiction of Watson–Crick base-paring (left: T-A and right: C-G); B) parallel triple helices: pyrimidine-rich third strand interactions are stabilised by Hoogsteen hydrogen bonds (the duplex is in black, TFO is in blue, Watson–Crick base-paring is shown with dashed bonds, and Hoogsteen base-paring is shown with hashed bonds). The relative orientation of phosphodiester backbones is indicated by the symbols "
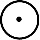
" and "
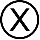
".

The ability to detect and modify the genome of living organisms is important for the diagnosis, prevention, and treatment of many diseases [1]. The site-specific targeting and manipulation of genomic DNA or RNA using chemically modified short oligodeoxynucleotides (ONs) is considered to be a viable therapeutic strategy [2–5]. Antigene strategies use ONs to specifically bind native DNA, induce genomic changes, and/or interfere with gene expression. Apart from strategies that use modular enzymes such as zinc-finger nucleases [6] or transcription activator-like effector nucleases (TALENs) [7] to recognise and cut DNA sequences, or CRISPR-CAS9 [8–10] and CAS9-constructs [11–14] which rely on large proteins to open the target duplex, triplex-forming oligonucleotides (TFOs) [15] can be designed to bind in a sequence-specific manner to double-stranded DNA (dsDNA) [16]. In forming the parallel triple-helix structure, a polypyrimidine TFO binds to dsDNA through Hoogsteen base-pairing [17], in which the cytosine bases in the TFO are protonated at the N3 atom (Figure 1B).

In antisense strategies, antisense ONs (AOs) interact with RNA molecules to interfere with protein expression [18–19]. The major challenge in designing chemically modified ONs as antigene/antisense agents is to ensure an efficient cellular uptake and nuclease resistance while still maintaining, or ideally increasing, binding affinity and specificity of the ONs towards their DNA or RNA target.

Many synthetic analogues of natural ONs, such as peptide nucleic acids (PNA) [20], locked nucleic acids [21] (LNA, also known as bridged nucleic acids (BNA) [22]) and phosphorothioate (PS) ONs [23–24] have been evaluated for antigene/antisense applications, however, each of the analogues did not meet all the requirements. For example, both PNA and modified PNAs have excellent chemical stability, are resistant to enzymatic degradation, and have high binding affinity towards complementary DNA and RNA, but have a tendency to aggregate, require high salt conditions, and have low solubility in water [1,25–26]. LNA (BNA) have an enhanced thermal stability in DNA triplexes and duplexes, a high binding affinity to RNA, and are nuclease resistant [22,26–28]. These properties have led to LNA (BNA) being used in various therapeutic ONs that have reached clinical trials [29]. However, the multistep synthesis of LNA and increased hepatotoxicity of some modified AOs ensure that further optimisation is required [30]. Chemical modification of ONs with a PS linkage resulted in ONs resistant to nuclease degradation but with several side effects due to nonspecific interactions with cellular components [31].

Modifications of the phosphate backbone of DNA and RNA, especially charge neutral modifications, have gained attention in recent years because such modifications not only improve the nuclease resistance of ONs but also enhance their affinity towards complementary DNA/RNA/dsDNA and improve cell uptake. The lack of a negatively charged backbone also improved the binding of PNA to DNA or RNA strands. It has been shown that positively charged PNA bind more strongly to DNA and RNA than negatively charged PNA at low salt concentrations (0–100 mM Na^+^) whereas at medium to high salt concentrations (250–1000 mM Na^+^) the trend is reversed [32].

As a charge-neutral phosphate mimic, the methylphosphonate linkage (PMe) has been introduced into the DNA backbone to improve stability of ONs towards enzymic digestion as well as DNA duplex and triplex binding affinity [33]. However, the poor aqueous solubility [34], reduced binding affinity with complementary RNA [35], and a destabilising effect on the thermal stability of G-quadruplexes [36] hinders its application. In contrast, a phosphate methylated linkage (POMe,) marginally destabilised complementary DNA but improved sequence specificity [37].

**Scheme 1:**
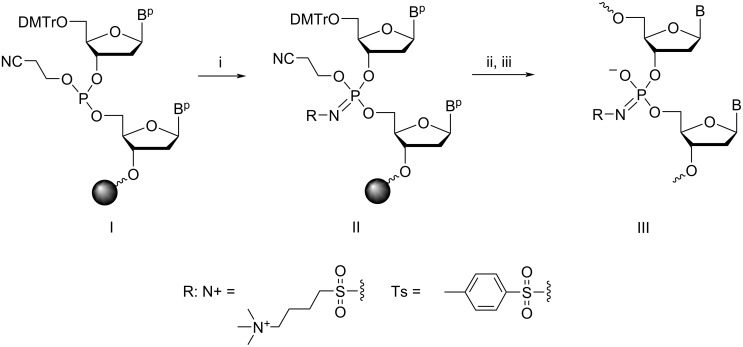
The synthesis of ONs with Ts and N+ modification using the Staudinger reaction during the solid-phase DNA synthesis. Conditions: (i) 0.5 M TsN_3_, MeCN, 37 °C, 30 min for Ts modification; 0.7 M 4-(azidosulfonyl)-*N,N,N*-trimethylbutan-1-aminium iodide, DMF, 37 °C, 30 min for the N+ modification; (ii) DNA synthesis; (iii) conc. aq NH_3_, 55 °C, 12 h; B^p^/B: protected/deprotected heterocyclic base; DMTr: 4,4'-dimethoxytrityl.

Recently, we synthesised a G-rich ON (TG_4_T) with all phosphates replaced by a 4-(trimethylammonio)butylsulfonyl phosphoramidate group (N+, Scheme 1). The sequence was designed to obtain the formally charge-neutral zwitterionic N+TG_4_T [38]. Each negatively charged phosphoramidate is neutralised by the positively charged quaternary ammonium group, providing a zwitterionic phosphate mimic. The resistance to enzymatic degradation, a higher thermal stability, and a faster association that was independent of ionic strength was observed for this N+-modified G-quadruplex (TG_4_T)_4_. These properties encouraged us to evaluate the N+ modification in the context of DNA duplexes and triplexes and to perform cell-uptake studies. For comparison, we also evaluated the properties of ONs modified with a tosyl sulfonyl phosphoramidate (Ts) that results in a negatively charged phosphate mimic [39].

The introduction of each, N+ or Ts modification creates a chiral center at the phosphorus atom resulting in a mixture of 2*^n^* diastereomers, where *n* is the number of modified phosphate groups. The reverse-phase (RP) HPLC purification occasionally results in the separation of individual diastereomers (usually for ONs with a single modification), which supports the insignificant difference in the lipophilicity of the diastereomeric ONs. We hypothesised that, in comparison with native ONs, the N+ONs should hybridise with higher affinity to complementary single-stranded DNA (ssDNA) or RNA due to both a reduced repulsion between negatively charged phosphates and a thermal stability being less dependent on the ionic strength of the solution. Moreover, N+ONs carrying zwitterionic phosphates could lead to an increased cell uptake.

For both N+ and Ts modifications, we synthesised 14-mer ONs with either one, two, three, or four modifications introduced at various positions in the sequence. The thermal stability of a parallel DNA triplex and duplexes of DNA and RNA formed with these ONs where then evaluated. Thermal denaturation experiments, nuclease resistance and cell-uptake assays were also conducted to evaluate these chemically modified ONs.

## Results

### Synthesis and purification of modified ONs

4-(Azidosulfonyl)-*N,N,N*-trimethylbutan-1-aminium iodide [38] and tosyl azide (*p*-toluenesulfonyl azide, TsN_3_) [39] were synthesised and used for the synthesis of the modified ONs using an automated DNA synthesiser as described. The solution of sulfonyl azide (0.5 M TsN_3_ in MeCN or 0.7 M 4-(azidosulfonyl)-*N,N,N*-trimethylbutan-1-aminium iodide in DMF) was introduced as replacement of a standard iodine/pyridine oxidation step to react with 3',5'-dinucleoside β-cyanoethyl phosphites (Scheme 1, I), forming the *N*-modified iminophosphorane (Scheme 1, II). ONs bearing one or more *N*-modified phosphoramidate groups (Scheme 1, III) were obtained after the removal of the protective groups and β-cyanoethyl groups using ≈28% ammonia. We found out that a higher conversion was observed when performing the Staudinger reaction at 37 °C rather than at room temperature. The yield was also improved by minimising the handling of the solid support and performing the reaction using a microtube pump to deliver the sulfonyl azide solution onto the column with CPG support. The cleaved and deprotected N+- and Ts-ONs were initially purified using reversed-phase (RP) HPLC. However, the separation of ONs with varying numbers of modifications was not ideal as there were only marginal changes in the retention time in RP-HPLC. Therefore, ion-exchange (IE) HPLC was used for purifying these ONs. The substitution of each phosphate with N+ modification, resulted in a shorter retention time (τ) in IE-HPLC (Δτ = −3 min/modification, Table 1). For Ts-modified ONs, the incorporation of the Ts modifications, as a result of increased hydrophobicity, led to an increased retention time (Δτ = +1.5 to +2 min/modification, Table 1) compared to the native sequence. The composition of the ONs was confirmed by electrospray ionization mass spectrometry (ESIMS) in the negative mode (Table 1). For clarity, we introduced the following nomenclature of the ONs synthesised. The prefix 5ʼ- or 3ʼ- with either N+ or Ts- means that the first phosphate at the 5ʼ- or 3ʼ-end was modified; m-N+ or m-Ts- indicates that the named modification was incorporated in the middle of the sequence; 2N+, 3N+, 4N+ or 2Ts-, 3Ts-, 4Ts- indicates that two, three, or four modifications were distributed evenly in the sequence.

**Table 1:** Names of the ONs synthesised, their sequences, retention times on the ion-exchange column^a^, compositions, and isolated yields.

	sequence	retention time (min)	calculated MW	observed MW^b^	isolated yield (%)

**ON1**	5'-CCCCTTTCTTTTTT^c^	31.53	4121.7	–	
**5ʼ-N+ON2**	5'-C**_N+_**CCCTTTCTTTTTT	27.76	4296.7958	4297.7588^d^	9^e^
**m-N+ON3**	5'-CCCCTTT**_N+_**CTTTTTT	27.43	4296.7958	4297.7455^d^	11^e^
**3ʼ-N+ON4**	5'-CCCCTTTCTTTTT**_N+_**T	27.74	4296.7958	4297.7466^d^	20^f^
**2N+ON5**	5'-C**_N+_**CCCTTTCTTTTT**_N+_**T	23.42	4473.9012	4473.8248^d^	8^e^
**3N+ON6**	5'-C**_N+_**CCCTTT**_N+_**CTTTTT**_N+_**T	20.77	4649.0067	4650.1412^d^	10^e^
**4N+ON7**	5'-C**_N+_**CCCT**_N+_**TTCT**_N+_**TTTT**_N+_**T	17.09	4826.0965	4826.0516^d^	23^f^
**4N+{FAM}**	5'-C**_N+_**CCCT**_N+_**TTCT**_N+_**TTTT**_N+_**T{FAM}	–^g^	5396.4012	5396.1460^h^	20
**5ʼ-Ts-ON8**	5'-C**_Ts_**CCCTTTCTTTTTT	32.80	4272.7151	4274.6664^d^	4^e^
**m-Ts-ON9**	5'-CCCCTTT**_Ts_**CTTTTTT	32.75	4272.7151	4273.4728^d^	5^e^
**3ʼ-Ts-ON10**	5'-CCCCTTTCTTTTT**_Ts_**T	32.81	4272.7151	4273.4694^h^	8^e^
**2Ts-ON11**	5'-C**_Ts_**CCCTTTCTTTTT**_Ts_**T	35.67	4425.7398	4427.7401^h^	6^e^
**3Ts-ON12**	5'-C**_Ts_**CCCTTT**_Ts_**CTTTTT**_Ts_**T	36.76	4578.7646	4578.6076^i^	30^f^
**4Ts-ON13**	5'-C**_Ts_**CCCT**_Ts_**TTCT**_Ts_**TTTT**_Ts_**T	40.07	4731.7893	4731.7620^j^	39^f^
**4Ts-{FAM}**	5'-C**_Ts_**CCCT**_Ts_**TTCT**_Ts_**TTTT**_Ts_**T{FAM}	–^g^	5301.2398	5302.8380^h^	26
**m-N+ON14**	5'-CCCCTTTCTTT**_N+_**TTT	27.50	4296.7958	4296.7520^d^	19^f^
**m-N+ON15**	5'-CCCCTTT C**_N+_**TTTTTT	27.43	4296.7958	4296.7300^d^	16^f^
**m-N+ON16**	5'-CCCC**_N+_**TTTCTTTTTT	27.45	4296.7958	4296.7440^d^	17^f^
**m-N+ON17**	5'-CC**_N+_**CCTTTCTTTTTT	27.62	4296.7958	4296.7350^d^	21^f^
**3N+ON18**	5'-C**_N+_**CCCTT**_N+_**TCTTTTT**_N+_**T	20.44	4649.0067	4649.937^d^	17^f^

^a^IE-HPLC was performed on an IE-column (TSKgel Super Q-5PW) using a gradient of NaCl concentration (0 → 0.5 M) in 20 mM Tris-HCl, 1 mM EDTA, pH 9.0 over 30 min; ^b^based on ESIMS in the negative mode; ^c^obtained from Integrated DNA Technologies; ^d^calculated for [M − 6H]^6−^; ^e^synthesised in a 1 µmol scale using a previously reported procedure with transferring the solid support from a column into a vial for reaction with sulfonyl azide for 30 min at room temperature. Afterwards, the solid support was transferred back to the column to continue DNA synthesis [39]. Some amount of the solid support was lost during the transfer and washing steps, especially for multiple modifications, which was the main reason for the low yields of these ONs. ^f^synthesised in a 1 µmol scale following a modified procedure using a microtube pump to deliver the sulfonyl azide solution onto the column with CPG-support at 37 °C [38]; ^g^synthesised in a 3–4 µmol scale, purified by 20% denaturing PAGE (7 M urea), followed by extraction from the gel and desalting. ^h^for [M − 7H]^5−^; ^i^for [M + K^+^ − 9H]^8−^; ^j^for [M − 4H]^4−^; the ESIMS spectra are provided in Supporting Information File 1.

The solubility of the ONs was not influenced by the introduction of Ts and N+ modifications, as the purified, desalted, and lyophilised ONs were fully dissolved in 50 µL H_2_O. The Ts-modified ONs have previously been shown to marginally destabilise duplexes with complementary DNA and RNA [39]. The chemical stability of **5ʼ-N+ON2** at various pH (5.5, 7.0, and 8.5) was evaluated by incubation in 10 mM Na phosphate buffer (140 mM NaCl, 0.1 mM Na_2_-EDTA) at 50 °C for 24 h. No degradation was observed according to IE-HPLC analysis (see Figure S16 in Supporting Information File 1), which ensures that the N+-modified ONs will be chemically stable during the evaluation of the thermal stability of complexes with complementary DNA and RNA.

### Thermal denaturation experiments

The thermal stability of antiparallel ON/RNA and ON/DNA duplexes as well as parallel DNA triplexes was assessed in thermal denaturation experiments and the results are summarised in Table 2.

**Table 2:** *T*_m_ [°C, ± 0.5 °C] data for triplex and duplex melting, taken from UV melting curves (λ = 260 nm).

entry		antiparallel duplex			triplex^c^	
				
		RNA^a^	DNA^b^			
		pH 7.0	pH 5.0	pH 7.0	pH 5.0^d^	pH 6.0

1	**ON1**	46	48	50	45	28
2	**5ʼ-N+ON2**	53 (+ 7.0)	44 (−4.0)	51 (+1.0)	40 (−5.0)	25 (−3.0)
3	**m-N+ON3**	47 (+ 1.0)	43 (−5.0)	48 (−2.0)	55 (+10.0)	28
4	**3ʼ-N+ON4**	58 (+ 12.0)	46 (−2.0)	52 (+2.0)	56 (+11.0)	29 (+1.0)
5	**2N+ON5**	53 (+ 7.0)	44 (−4.0)	52 (+2.0)	56 (+11.0)	28
6	**3N+ON6**	41 (− 5.0)	45 (−3.0)	51 (+1.0)	48 (+3.0)	<15
7	**4N+ON7**	55 (+ 9.0)	48 (0.0)	51 (+1.0)	48 (+3.0)	28
8	**5ʼ-Ts-ON8**	54 (+ 8.0)	39 (−9.0)	46 (−4.0)	51 (+6.0)	24 (−4.0)
9	**m-Ts-ON9**	44 (−2.0)	31 (−17.0)	37 (−13.0)	51 (+6.0)	<15
10	**3ʼ-Ts-ON10**	57 (+11.0)	44 (−4.0)	49 (−1.0)	54 (+9.0)	27 (−1.0)
11	**2Ts-ON11**	56 (+10.0)	43 (−5.0)	51 (+1.0)	53 (+8.0)	25 (−2.0)
12	**3Ts-ON12**	38 (−8.0)	40 (−8.0)	45 (−5.0)	49 (+4.0)	<15
13	**4Ts-ON13**	53 (+7.0)	39 (−9.0)	44 (−6.0)	47 (+2.0)	20 (−8.0)
14	**m-N+ON14**	54 (+8.0)	–^e^	51 (+1.0)	–	–
15	**m-N+ON15**	56 (+10.0)	–	51 (+1.0)	–	–
16	**m-N+ON16**	54 (+8.0)	–	55 (+5.0)	–	–
17	**m-N+ON17**	56 (+10.0)	–	52 (+2.0)	–	–
18	**3N+ON18**	54 (+8.0)	–	51 (+1.0)	–	–

^a^The RNA sequence for the antiparallel duplex formation is **ON19**: 3'-rGGGGAAAGAAAAAA; *c* = 1.0 µM of each strand in 20 mM sodium cacodylate, 100 mM NaCl, 10 mM MgCl_2_, pH 7.0; the *T*_m_ values for the ON/RNA duplexes were confirmed by CD melting experiments (Figures S7 and S8, and Table S2 in Supporting Information File 1); ^b^the DNA sequence for the antiparallel duplex formation is **ON20**: 3'-GGGGAAAGAAAAAA; *c* = 1.0 µM of each strand in 20 mM sodium cacodylate, 100 mM NaCl, 10 mM MgCl_2_, pH 5.0 and pH 7.0; ^c^*c* = 1.5 µM of **ON1–13** and 1.0 µM of each strand of dsDNA (**D1**: 3'-CTGCCCCTTTCTTTTTT/5'-GACGGGGAAAGAAAAAA) in 20 mM sodium cacodylate, 100 mM NaCl, 10 mM MgCl_2_, pH 5.0, 6.0 and 7.0; duplex *T*_m_ = 56.5 °C (pH 5.0), 58.5 °C (pH 6.0), and 57.0 °C (pH 7.0); triplex formation was confirmed by size-exclusion HPLC (SE-HPLC) in sodium cacodylate buffer (pH 5.0 and pH 6.0, Figure S15 in Supporting Information File 1), no triplex was formed at pH 7.0; ^d^the *T*_m_ for triplex melting was determined by subtraction of the duplex melting curve from the overlaid melting curve (Figure S6 in Supporting Information File 1); ^e^not performed.

The sequences possessing a different number of N+ and Ts modifications were studied initially in an antiparallel duplex formed with complementary RNA and compared with the corresponding antiparallel DNA duplexes at pH 7.0. Apart from the ONs possessing modifications in the middle of the sequence (entries 6, 9, and 12 in Table 2), stabilised ON/RNA duplexes were obtained for both N+ONs (Δ*T*_m_ = +1 – +12 °C, entries 2–7, Table 2) and Ts-ONs (Δ*T*_m_ = +7 to +11 °C, entries 8–13, Table 2). The highest thermal stabilisation against RNA induced by a single modification was observed for ONs with one modification at 3ʼ-end (Δ*T*_m_ = +12 °C and +11 °C for N+ and Ts modifications, respectively). The corresponding antiparallel DNA duplexes were less thermally stable with Δ*T*_m_ = −1 to +2 °C. The same trend was seen for ONs with a modification at both the 5ʼ- and 3ʼ- ends: ON/RNA duplexes were more stable (Δ*T*_m_ = +7 °C for **2N+ON5** and Δ*T*_m_ = +10 °C for **2Ts-ON11**) than the corresponding antiparallel DNA duplexes (Δ*T*_m_ = +2 °C for **2N+ON5** and Δ*T*_m_ = +1 °C for **2Ts-ON11**). The thermal stability of the ON/RNA duplexes was not improved by increasing the number of N+ or Ts modifications in the ONs.

For Watson–Crick-type duplexes, both the N+ and Ts modifications destablised the DNA duplex at pH 5.0. The destablising effect was more pronounced for Ts than for the N+ modification (Δ*T*_m_ = −17 to −4 °C for Ts and −5 to 0 °C for N+ modifications, respectively). For the antiparallel ON/DNA duplexes formed at pH 7.0, when comparing ONs with the same number of modifications, the N+ modifications led to higher *T*_m_ values than Ts-ONs. The incorporation of three and four Ts moieties led to a further decrease in the *T*_m_ whereas the corresponding N+ONs did not disrupt the duplex thermal stability. These results indicate that the N+ and Ts modifications can be viewed as RNA-like modifications, because their use in ONs led to higher Δ*T*_m_ values for ON/RNA than for ON/DNA duplexes.

The same modified sequences (**ON1**–**13**) were also studied in a pH-dependent Hoogsteen-type base-pairing towards the duplex **D1** forming a parallel triplex [40]. As can be seen in Table 2, all parallel triplexes formed at pH 5.0 were more thermally stable than at pH 6.0 and no triplex was formed at pH 7.0, which is consistent with the trend for parallel triplexes based on CT-TFOs [41]. Some fluctuations were observed for Hoogsteen-type triplexes formed by N+ONs. A modification at the 5’ end destabilised triplexes at both pH 5.0 and 6.0 (Δ*T*_m_ = −5 °C and −3 °C, respectively, Table 2). All other N+ONs formed more stable triplexes with **D1** at pH 5.0, while marginal changes were observed for triplexes at pH 6.0 except for **3N+ON6** with three modifications that did not form a triplex. The incorporation of Ts modifications led to stabilised Hoogsteen-type triplexes at pH 5.0 (Δ*T*_m_ = +2 to +9 °C, Table 2), whereas triplexes at pH 6.0 were less stable (Δ*T*_m_ = −1 to −8 °C). For Ts-ONs with the modification in the center of the sequence (**m-Ts-ON9** and **3Ts-ON12**), no triplex formation was observed at pH 6.0/room temperature. These results show that Hoogsteen-type triplexes with single N+ or Ts modifications at the 3ʼ-end are more thermally stable at the 5ʼ-end, and that increasing the number of modifications showed no advantage for *T*_m_ of parallel triplexes.

A position-dependent influence of the N+ and Ts moieties on the *T*_m_ is suggested by the less thermally stable duplexes formed by the ONs with a single modification in the middle of the sequence (in TC motif) compared to native DNA. We synthesised another set of N+ONs, with single (**ON14–17**, entries 14**–**17, Table 2) and triple modifications (**ON18**, entry 18, Table 2) that had no modifications in the center of the sequence and evaluated the thermal stability of their antiparallel duplexes formed with complementary RNA and DNA at pH 7.0. The results in Table 2 show that **ON14–17** form more stable duplexes with RNA (Δ*T*_m_ = +8 to +10 °C) and DNA (Δ*T*_m_ = +1 to +5 °C). It is interesting that sequences with the N+ modification in the CT motif (**m-N+ON15** and **m-N+ON16**) did not destabilise the antiparallel duplexes unlike the N+ modification in the TC motif (**m-N+ON3** and **3N+ON6**). One possible reason for this position-dependent influence of the N+ and Ts moieties on the duplex stability might be due to a propeller twist [42] in the TC dinucleotide interfering with the N+ and Ts moieties and destabilising the DNA and RNA duplexes.

Next, we evaluated the binding affinity of the N+ and Ts-modified ONs for complementary DNA and RNA at different salt concentrations (25, 50, and 100 mM NaCl, Table 3). It has been reported that the thermal stability of DNA duplexes decreases as salt concentrations are reduced due to the increased electrostatic repulsion between the negatively charged phosphates [43]. The native DNA duplex **ON1**/**ON20** showed a decline in the *T*_m_ values from 50 to 37 °C and to 19 °C, when the NaCl concentration was decreased from 100 to 50 mM and to 25 mM, respectively (Table 3). A similar trend was observed for the Ts-modified ONs as the backbone is still negatively charged. In contrast, for the N+ONs the decrease in the *T*_m_ with decreasing NaCl concentration was not as significant as for the negatively charged ONs, and the *T*_m_ value at a 25 mM salt concentration was 20 °C higher than the *T*_m_ for the unmodified duplex, and 12 °C higher than the Ts-modified duplex. However, such behaviour was not as noticeable for the duplexes formed with complementary RNA. The *T*_m_ value of the control ON/RNA duplex (**ON1/ON19**) decreased by 2 °C when the NaCl concentration was reduced from 100 to 25 mM, whereas the *T*_m_ for the N+ON/RNA duplex (**4N+ON7**/**ON19**) decreased by 10 °C, although it was still more thermally stable than the control ON/RNA duplex. In contrast, the duplex formed by Ts-ON and RNA (**4Ts-ON13**/**ON19)** was destabilised (Δ*T*_m_ = −6 °C) at the lowest salt concentration tested.

**Table 3:** The *T*_m_ [°C] and thermodynamic data at 298 K for the antiparallel duplexes at different NaCl concentrations, taken from UV melting curves (λ = 260 nm).^a^

	antiparallel duplex	NaCl (mM)	*T*_m_ (°C)^b^	Δ*H*^c^ (kJ/mol)	*T*Δ*S* (kJ/mol)	Δ*G*_298_ (kJ/mol)

ON/DNA	**ON1/ON20**	25	19	−430 (±20)	−400 (±20)	−30 (±28)
		50	37	−400 (±9)	−350 (±8)	−50 (±12)
		100	50	−368 (±8)	−305 (±7)	−63 (±10)
	**4N+ON7/ON20**	25	41 (+22.0)	−388 (±9)	−334 (±8)	−54 (±12)
		50	44 (+7.0)	−322 (±15)	−266 (±14)	−56 (±20)
		100	51 (+1.0)	−320 (±8)	−260 (±7)	−60 (±10)
	**4Ts-ON13/ON20**	25	29 (+10.0)	−382 (±14)	−340 (±14)	−42 (±19)
		50	34 (−3.0)	−372 (±12)	−326 (±11)	−46 (±16)
		100	45 (−5.0)	−354 (±7)	−297 (±7)	−57 (±10)
ON/RNA	**ON1/ON19**	25	44	−390 (±10)	−332 (±9)	−58 (±13)
		100	46	−306 (±17)	−248 (±17)	−58 (±24)
	**4N+ON7/ON19**	25	45 (+1.0)	−419 (±7)	−359 (±6)	−60 (±9)
		100	55 (+9.0)	−329 (±13)	−264 (±12)	−66 (±17)
	**4Ts-ON13/ON19**	25	38 (−6.0)	−420 (±20)	−370 (±20)	−50 (±28)
		100	53 (+7.0)	−407 (±13)	−337 (±12)	−70 (±17)

^a^One µM of each strand in 20 mM sodium cacodylate buffer (pH 7.0, supplemented with 25, 50, or 100 mM NaCl, respectively); ^b^*T*_m_ values are reported with ±0.5 °C uncertainties as determined from several experiments; values in parentheses are Δ*T*_m_ values calculated as *T*_m_ (sample) − *T*_m_ (unmodified duplex) at the same salt concentration; ^c^thermodynamic parameters are calculated as described in Supporting Information File 1 (see also Figures S9–S14) at 298 K, errors were calculated as described in reference [44].

We analysed the melting profiles and obtained the thermodynamic parameters of the duplexes at different salt concentrations ignoring the changes in DNA and salt concentrations induced by solution evaporation, the change of pH during heating, and assuming that there is no change in the heat capacity (Δ*c*_p_ = 0) [45]. We also assumed a two-state transition between duplex and single-stranded DNA and a linear relationship between the CD/UV signal and fraction of molecules unfolded (see Supporting Information File 1 for the analysis of the melting curves). As the thermal stability of DNA duplexes increased with increasing concentrations of salt, we expected a favourable Δ*H* of duplex formation at a higher salt concentration. However, in all cases studied, Δ*H* was less favourable at 100 mM than at 25 mM NaCl. Recently, similar observations have been reported for DNA modified with methyl phosphotriester linkage (POMe) using isothermal titration calorimetry (ITC) measurements which provides Δ*H* values directly [37].

For the native DNA duplex (**ON1/ON20**), the more favourable Δ*H* at the low salt concentration (25 mM NaCl) was deprived by an even higher entropy penalty leading to a loss in Δ*G* (ΔΔ*G*_298_ = 33 kJ/mol), thus lowering the *T*_m_ value at 25 mM NaCl. For the unmodified RNA duplex (**ON1/ON19**), ΔΔ*H* between 25 mM and 100 mM NaCl is 84 kJ/mol, and the corresponding entopic factor Δ(*T*Δ*S*) is 84 kJ/mol. As a result, changes in Δ*G*_298_ were negligible as reflected by the small decrease in the *T*_m_ value (Δ*T*_m_ = 2 °C) when the salt concentration was reduced from 100 mM to 25 mM NaCl.

For the N+ or Ts-modified ONs, Δ*H* for ON/DNA duplexes was less favourable at the same salt concentration than for the unmodified duplex, whereas *T*Δ*S* was more favourable. According to Kuo et al. [37], an increase in the salt concentration stabilises the DNA duplex by reducing the entropy costs of duplex formation rather than by reducing the strand charge repulsion. This reduction of entropy costs for duplex formation is due to the endothermic release of DNA-hydrating ordered water molecules into the bulk solvent. Since the introduction of the N+ modification compensated for the negative charge on the DNA backbone, the change in the entropy costs for N+ON/DNA between 100 mM and 25 mM NaCl solutions was less than that for the unmodified duplex (Δ(*T*Δ*S*) = −74 kJ/mol for N+ON/DNA versus −95 kJ/mol for the native DNA duplex, respectively). A similar trend was seen for the Ts-modified ONs (Δ(*T*Δ*S*) = −43 kJ/mol), but the change in Δ*H* between 25 mM and 100 mM NaCl was the lowest for the duplexes with DNA (ΔΔ*H* = 28 kJ/mol). This indicates that the hydrophobicity of Ts results in less water molecules involved in the formation of hydrogen bonds with dsDNA. However, it does not improve the interaction between two DNA strands, possibly due to the large size of the Ts moiety.

For duplexes of the N+ and Ts-ONs formed with complementary RNA, both Δ*H* and *T*Δ*S* terms were more negative at the same salt concentration than those for the unmodified ON/RNA complex, which is the opposite to duplexes formed with DNA. This led to even larger enthalpy–entropy compensation at the medium salt concentration (100 mM NaCl). When the NaCl concentration decreased from 100 mM to 25 mM, even though the reduction of entropy costs for the Ts-ON/RNA duplex was minimal for the RNA (Δ(*T*Δ*S*) = −33 kJ/mol), the loss in Δ*G* for Ts-ON/RNA duplexes was larger than that of unmodified and N+ON/RNA duplexes (ΔΔ*G*_298_ = 0, 6, and 20 kJ/mol for unmodified DNA/RNA, N+ON/RNA, and Ts-ON/RNA, respectively). This resulted in an unstable Ts-ON/RNA duplex at 25 mM NaCl. However, it should be noted that a significantly improved Δ*H* for Ts-ON/RNA duplex in comparison with the native ON/RNA at 100 mM NaCl (Δ*H* = −407 kJ/mol versus −306 kJ/mol, respectively) which is accountable for the more favourable Δ*G*_298_ and higher *T*_m_ value.

### Evaluation of N^+^ and Ts-modified ONs towards enzymatic digestion

**Figure 2:**
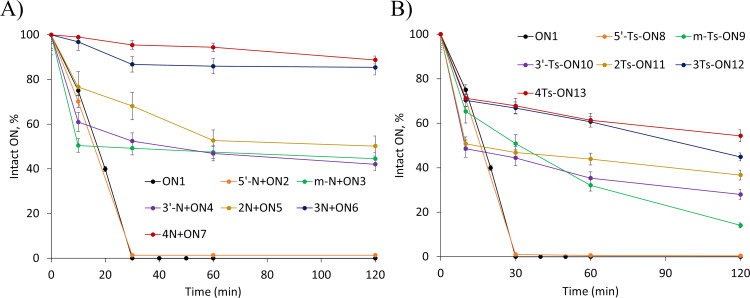
Percentage of intact ONs after 120 min. A) N+ONs; B) Ts-ONs. Percentage of intact ONs was determined by the ratio of full-length ONs at individual time in comparison with the sample at 0 min.

The nuclease resistance of the modified ONs was evaluated using snake venom phosphodiesterase (phosphodiesterase I, Sigma) and compared to the unmodified sequence **ON1**. Under the conditions used in this experiment, **ON1** was completely degraded within 30 min (Figure 2). Both, N+ and Ts-modified ONs showed an enhanced nuclease resistance when modifications were present at the 3ʼ-end and /or in the middle of the sequence. A single N+ or Ts modification at the 5ʼ-end of the ON did not provide protection against phosphodiesterase I. However, the resistance of the modified ONs towards phosphodiesterase increased with the number of modifications present. N+ONs, with the same number of modifications, showed a higher resistance to nuclease degradation than Ts-ONs. For example, 92.0 ± 1.8% of **4N+ON7** remained intact, whereas only 54 ± 3% of **4Ts-ON13** was intact after 120 min of enzymatic digestion (Figure 2, see also Figure S17 in Supporting Information File 1). N+ONs possessing more than four modifications showed a full enzymatic resistance after 120 min [38].

### Cell-uptake study

The cellular uptake of three modified ONs synthesised possessing four or five N+ or four Ts modifications and a fluorescent label (6-FAM) at the 3ʼ-end (Table 1) was tested. Asynchronously growing NIH3T3 mouse fibroblasts were incubated with the ONs for 12 hours, fixed in 4% paraformaldehyde before the cells were processed for fluorescent confocal microscopy.

**Figure 3:**
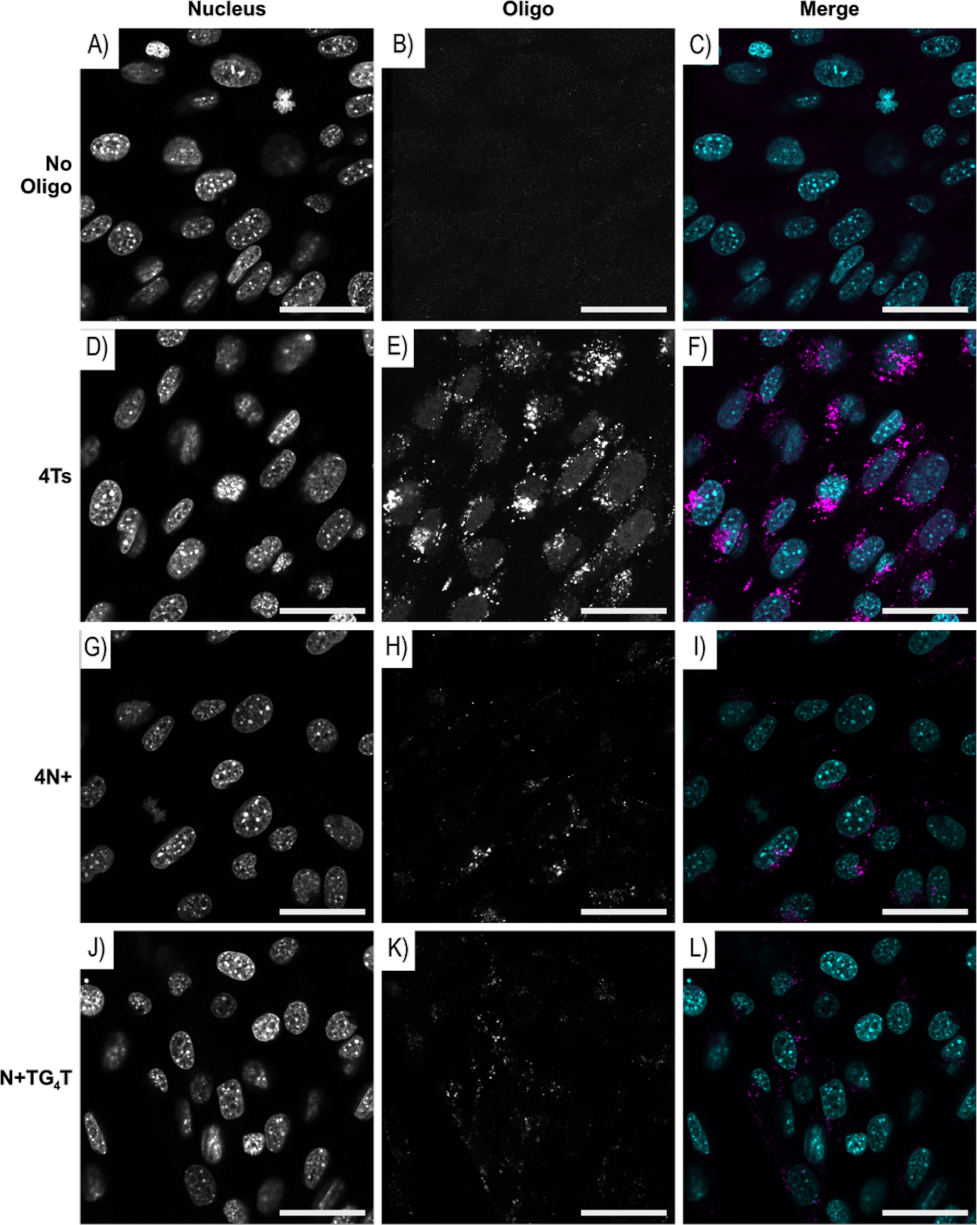
Representative images of mouse NIH 3T3 fibroblasts incubated with either (A–C) no oligo or 20 µM of (D–F) 4Ts, (G–I) 4N+, and (J–L) N+TG_4_T FAM labelled ONs. Asynchronously growing NIH3T3 cells were incubated for 12 hours with 20 µM of the stated FAM-labelled ONs or without ON, then fixed with 4% paraformaldehyde before staining with Hoechst 3342 to identify nuclear DNA. The images were collected with a Leica SP5 DM6000B scanning confocal microscope. Individual panels, nucleus/Hoechst 3342 and oligo/FAM are shown for each section, along with merge where pseudo-coloured panels are overlaid, nucleus (blue) and oligo (magenta). Scale bar: 40 μm.

**Figure 4:**
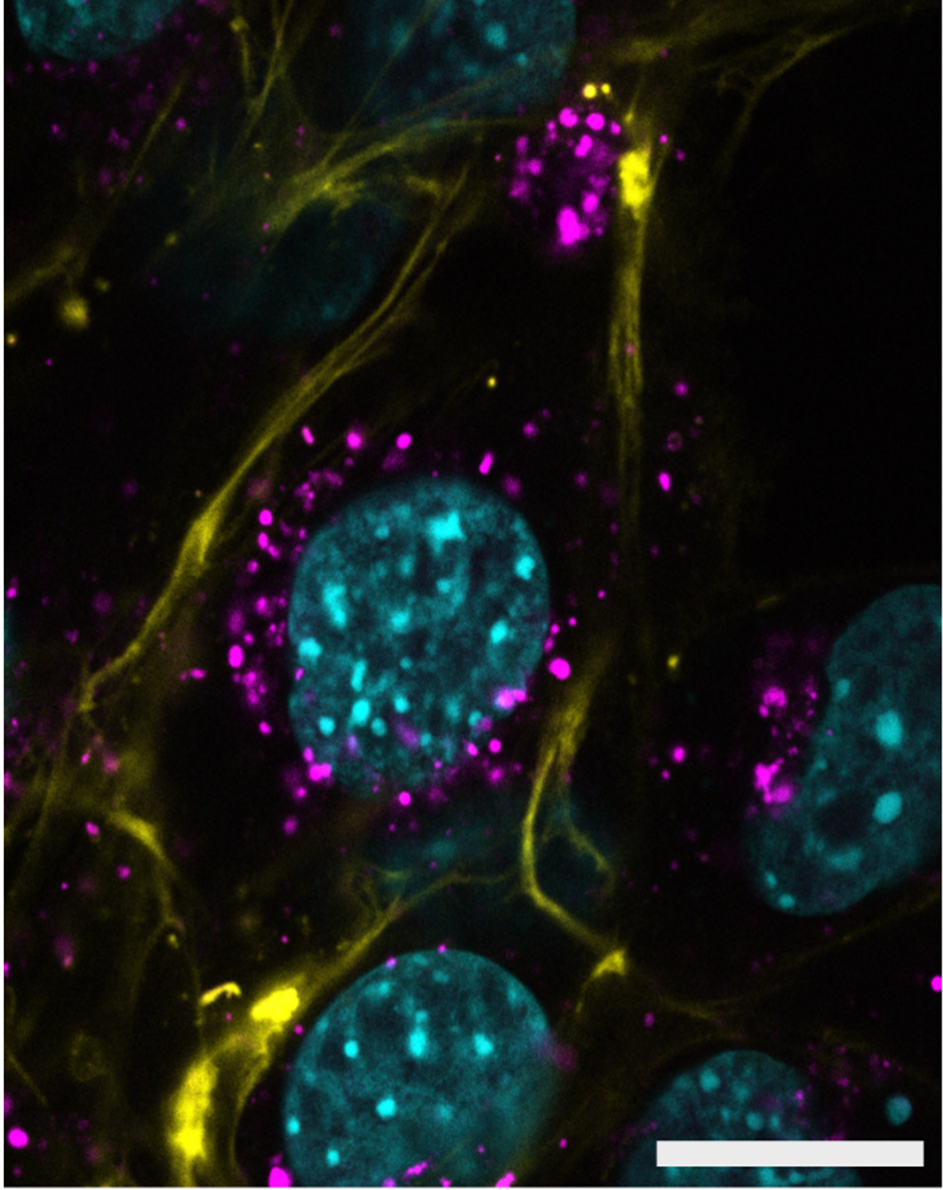
Representative confocal microscopy section showing the FAM vesicles inside the cell. Mouse NIH 3T3 fibroblasts were incubated with 20 µM of the FAM-labelled 4Ts-ON for 12 hours, then stained with CellBrite Fix 640 (Biotium) to identify the cell membrane before fixation with 4% paraformaldehyde. Nuclear DNA was then stained with Hoechst 3342 and images collected with a Leica SP5 DM6000B scanning confocal microscope. Overlaid pseudo-coloured panels of a section are shown: nuclear DNA (blue), 4Ts-FAM/ON (magenta), cell membrane (yellow). Scale bar: 20 μm.

Figure 3 shows that FAM-labelled Ts and N+-modified DNAs are concentrated in vesicles (punctate foci in the oligo/FAM panel) that accumulate around the edge of the nucleus. Interestingly, the Ts-modified oligo is also present in the nucleus as indicated by the colocalisation of the ON (Figure 3E) with the nuclear DNA (Figure 3D). The diffuse nuclear pattern of the Ts-modified ON suggests they can escape the endocytic vesicles and enter the nucleus via the nuclear pores. This is in contrast to the lack of colocalisation of the FAM signal with the nuclear DNA in the negative control (no oligo) and for the N+-modified oligos (Figure 3B, H, and K, respectively). Confocal microscope sections that dissect the nucleus were collected showing that the FAM-ON imaged were in the cytoplasm when localised adjacent to the nucleus and not on the cell surface. Staining of the cell membrane, along with the nuclear DNA, confirmed that the Ts-ON foci are present within the cytoplasm as shown in Figure 4.

## Discussion

Chemical modification provides an effective and efficient way of obtaining therapeutic antigene/antisense agents based on the nucleic acid scaffold. To regulate transcription or translation, chemically modified ONs need to be able to enter the cell, resist nuclease degradation, not be toxic to the cell, and importantly, bind to the target DNA or RNA in a sequence-specific manner with high affinity [46]. The electrostatic repulsion between negatively charged phosphates is considered to be one of the factors that determines the thermodynamic stability of nucleic acid secondary structures. Neutral or positively charged oligonucleotide analogues should bind more tightly with complementary DNA or RNA. Several studies have focused on the introduction of positively charged groups to a nucleobase [47–48], a sugar [49–50], or the DNA backbone [51–53] leading to the formation of more stable duplexes and triplexes [54]. The introduction of sulfonamide RNA (SaRNA monomers) to replace the phosphodiester backbone led to charge-neutral sulfonamide antisense oligonucleotides (SaASOs), which resulted in a lower destabilisation (a more stable) DNA–RNA duplex compared to a DNA–DNA duplex [55]. In contrast, the incorporation of branched, charge-neutralising sleeve (BCNS) groups onto the DNA backbone led to self-neutralising ONs that did not induce a change of *T*_m_ when binding complementary DNA sequences [56], regardless of the number of BCNSs incorporated. This is in line with our results where increasing the number of N+ or Ts modifications showed no benefit in *T*_m_ values of the antiparallel duplexes formed with complementary RNA or DNA.

In order to be compatible with standard automated solid-phase DNA or RNA synthesis, the introduction of SaRNA monomers into an RNA backbone involved a 14-step preparation of the phosphoramidite for the SaRNA-TT dinucleotide. Similarly, the incorporation of BCNS groups on a DNA backbone requires the synthesis of thymidine and 2’-OMe-uridine phosphoramidites bearing BCNS groups comprising nine and ten synthetic steps, respectively. In comparison, the synthesis of an N+ monomer requires only four synthetic steps starting from commercially available 1,4-butane sultone that does not have any silica gel purification [38]. The incorporation of the N+ modification onto DNA is performed during DNA synthesis instead of a standard oxidation step. Moreover, N+ and Ts modifications can be introduced into any position in the sequence, which is not the case for SaRNA and BCNS nucleic acid analogues.

Unlike PNAs and some of BCNS groups, N+ and Ts-modified ONs demonstrated excellent chemical stability and solubility in buffer solutions. The presence of the N+ modification enhanced the stability of parallel triplexes at pH 5.0. The Ts modification also stabilises parallel triplexes at pH 5.0, but the stability decreased with increasing number of Ts moieties incorporated. Apart from ONs with N+ or Ts modifications in the middle of the sequence, both types of modified ONs hybridised with complementary RNA with a higher thermal stability than with DNA, suggesting that the N+ and Ts modifications can be used in antisense strategies. This is in contrast with reports that ONs with Ts groups destablised the duplex formation with complementary RNA (Δ*T*_m_ = −1.6 to −1.2 °C/modification) [39], which suggests that the effect of the Ts modification on the *T*_m_ is dependent on the sequence.

The thermal stability of duplexes formed by N+ONs and their complementary DNA sequence was less dependent on the ionic strength, which was predicted for zwitterionic nucleotides that can bind to natural DNA at low ionic strength as well or better than natural DNA [57]. Similar results have been reported for ONs with BCNS groups: the *T*_m_ of 2’-OMe duplexes was increased with increasing numbers of BCNSs at low ionic strength (25 mM HEPES buffer, pH 7.3). When binding to complementary RNA sequences, such behaviour was not as noticeable. It has been reported in the past that *T*_m_ values for ON/RNA duplexes are less sensitive to changes in the ionic strength in comparison with ON/DNA duplexes [58]. The Ts modification stabilised the duplex formation with RNA at a salt concentration of 100 mM , but destabilised the RNA duplex at low salt concentration.

The thermodynamic analysis of the melting curves revealed that the N+ modification stabilises the duplex with DNA because of a significantly reduced loss in entropy but stabilises the duplex formation with RNA because of the improved enthalpy at the same salt concentrations. A similar trend for Δ*H* and *T*Δ*S* is observed for the Ts modification compared to native DNA.

In line with the recent report [37], the loss of the thermodynamic stability at low salt concertations for the native DNA duplex was caused by the large entropic penalty that was not compensated by the improved enthalpy. For the native RNA duplex, entropic penalty and improved enthalpy cancelled each other out resulting in a similar thermodynamic stability in the presence of 25 and 100 mM NaCl.

The polyelectrolyte ion condensation theory can be used to explain how an N+ modification stabilises duplex formation: For natural DNA, the double-helical form has a higher charge density in comparison with the single-stranded form. During denaturation, a portion of the counterions bound to DNA are lost to the bulk solvent due to the reduction in charge density. For a DNA duplex with one zwitterionic strand, the charge density of duplex and single stranded states is balanced, and only a fraction of the counterions should be lost during denaturation. As a result, the thermal stability of zwitterionic N+ DNA duplexes was less dependent on the ionic strength [57,59]. This is in line with our thermodynamic analysis that the dsDNA having N+ modifications showed less entropy costs when the ionic strength changed.

Native DNA and RNA sequences are highly susceptible to nuclease degradation within the cell. A modification on the phosphate group reduces the possibility of enzymatic digestion, which will be useful for cellular applications of N+ and Ts-modified ONs. The introduction of even a single N+ and Ts modification at the 3ʼ-end, but not at the 5ʼ-end, leads to the resistance of the modified ONs to enzymatic digestion by snake venom phosphodiesterase I.

The FAM-labelled ONs were shown to enter cells without the use of a transfection reagent. After a 12 hour incubation, the Ts-ON was present in both the cytoplasm and nucleus. In comparison, there was less cellular uptake of 4N+{FAM} ON and it was only present in the cytoplasm. These results indicate that ONs with phosphate modifications such as N+ or Ts might be suitable tools for the application of DNA and RNA vaccines [60], for the treatment of cancer [61], infectious diseases [62], and neurological disorders [63].

## Conclusion

ONs possessing N+ and Ts modifications have good aqueous solubility and chemical stability, which allowed the assessment of these modifications in the context of DNA triplexes and duplexes. The presence of N+ or Ts modifications on the internucleotidic phosphates enhanced the binding affinity of the ONs for complementary RNA and increased their resistance to digestion by phosphodiesterase I. Fluorescently labelled Ts-ONs penetrate the cell and enter the nucleus, while N+ONs remain trapped in vesicles in the cytoplasm. These properties make the N+ and Ts-modified ONs promising candidates for cell-based applications.

## Supporting Information

File 1 Experimental part.
