# Supplementary material for: DNA with zwitterionic and negatively charged phosphate modifications: Formation of DNA triplexes, duplexes and cell uptake studies
Source: Beilstein J Org Chem. 2021 Mar 29;17:749–61. doi: 10.3762/bjoc.17.65 (PMC8022206; doi:10.3762/bjoc.17.65)
Supplement: File 1 — Experimental part. [file Beilstein_J_Org_Chem-17-749-s001.pdf]

BEILSTEIN JOURNAL OF ORGANIC CHEMISTRY

Supporting Information

for

DNA with zwitterionic and negatively charged phosphate modifications: Formation of DNA triplexes, duplexes and cell uptake studies

Yongdong Su, Maitsetseg Bayarjargal, Tracy K. Hale and Vyacheslav V. Filichev

Beilstein J. Org. Chem. **2021,** *17,* 749–761. doi:10.3762/bjoc.17.65

Experimental part

License and Terms: This is a supporting information file under the terms of the Creative Commons Attribution License (https://creativecommons.org/licenses/by/4.0). Please note that the reuse, redistribution and reproduction in particular requires that the author(s) and source are credited and that individual graphics may be subject to special legal provisions.
The license is subject to the *Beilstein Journal of Organic Chemistry* terms and conditions: (https://www.beilstein-journals.org/bjoc/terms)

Table of content

1. Synthesis and purification of chemically modified ONs S2
2. ESIMS analysis of modified ONs S4
3. Determination of melting temperature of duplexes and triplexes using UV–vis spectrometry S8
   3.1. Determination of melting temperature using CD measurements S11
   3.2. Determination of thermodynamic parameters of antiparallel duplexes formed at different salt concentrations S13
4. Evaluation of triplex formation using size-exclusion HPLC S20
5. Evaluation of stability of 5’N+ON2 in Na-phosphate buffer at various pH at 55 °C S21
6. Enzymatic digestion of ONs by phosphodiesterase I S21
7. Cell culture assay S23
8. References S23

1. Synthesis and purification of chemically modified ONs

4-(Azidosulfonyl)-*N*,*N*,*N*-trimethylbutan-1-aminium iodide[1] and tosyl azide (*p*-toluenesulfonyl azide, TsN_3)[2] were synthesised as described and used for the synthesis of the modified ONs. One should note that TsN_3 should be handled with caution as it has the shock sensitivity of tetryl (*N*-methyl-*N*-2,4,6-tetranitroaniline) and the explosiveness of TNT[3].

Unmodified oligonucleotides (ONs) were purchased from Integrated DNA Technologies (IDT, Singapore). The N+ and Ts-modified ONs were synthesised using a Mermaid-4 automated DNA synthesiser (BioAutomation Corp.) using 5-ethylthio-1*H*-tetrazole (ETT) as an activator. Oxidation and deprotection times were 60 s (repeat 3 times) and coupling time 90 s for 5 μmol synthesis scale. When the modifications were incorporated, the automatic synthesis was paused before the capping step (after coupling step and wash), the column was taken out from the DNA synthesiser and connected to a micro tube pump. For N+ modification, 4-(azidosulfonyl)-*N*,*N*,*N*-trimethylbutan-1-aminium iodide in DMF (saturated and degassed, 2 mL) was pumped through the column for 30 min at 37 °C. For the Ts-modification, TsN_3 in MeCN (0.5 M, 2 mL) was pumped through the column for 30 min at 37 °C. The column was placed back in the synthesiser to continue the ON synthesis. The resulting ONs were cleaved from the solid support, and deprotected with concentrated aqueous ammonia (≈ 28%) at 55 °C for 12 h.

The ONs were purified by HPLC using IE-column (TSKgel Super Q-5PW). Buffer A (20 mM Tris-HCl, 1 mM Na_2-EDTA, pH 9.0), buffer B (20 mM Tris-HCl, 1 mM Na_2-EDTA, 1 M NaCl, pH 9.0). Gradients: 3.7 min 100% A, convex curve gradient to 30% B in 7.3 min, linear gradient to 50% B in 18.5 min, concave gradient to 100% B in 7.4 min, keep 100% B for 7.4 min and then 100% A in 7.4 min. Collected individual UV-absorbing fractions (λ = 260 nm) were desalted using a NAP-25 column. The composition of each fraction was confirmed by ESIMS (Table 1 and Figures S2–S5), and the fractions containing the desired modified ONs were identified and freeze-dried.

The purity of the chemically modified ONs was confirmed using 20% denaturing gel (Figure S1). Gels were prepared in TBE buffer (pH 8.0) in 7 M urea with 0.5 mm thickness, 17.5 × 14.5 cm^2 (19:1 acrylamide/bisacrylamide ratio). Samples were incubated at high temperature in 7 M urea (7.5 μL) to disrupt high order assemblies. TBE buffer (pH 8.0) was used as a running buffer. Gel electrophoresis was performed at 15 W per gel at 4 °C to avoid overheat. After the electrophoresis, gels were stained with 5% Stains-All® solution in water/formamide 1: 1 for 5–10 min and then destained in H_2O until complete fading of the dye from the gel background.

A)

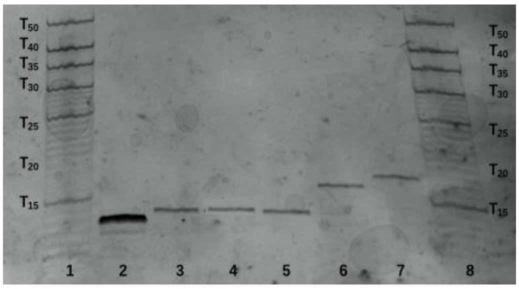

B)

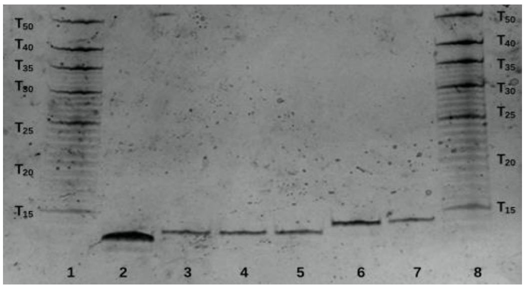

C)

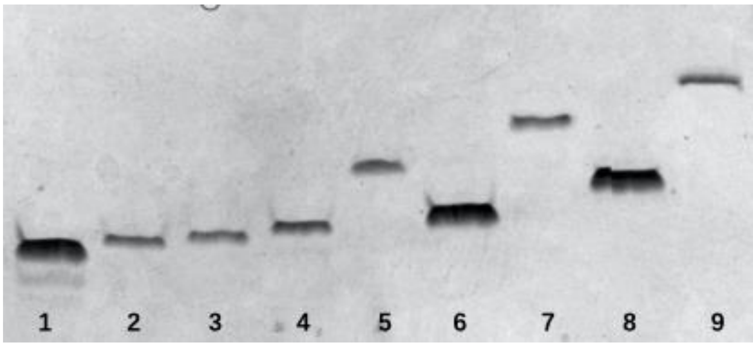

Figure S1: 20% Denaturing polyacrylamide gel (7M urea) of chemically modified ONs. A) Lanes are 1: ladder; 2: ON1; 3: **5’-N+ON2**; 4: **m-N+ON3**; 5: **3’-N+ON4**; 6: **2N+ON5**; 7: **3N+ON6**; 8: ladder. B) Lanes are 1: ladder; 2: **ON1**; 3: **5’-Ts-ON8**; 4: **m-Ts-ON9**; 5: **3’-Ts-ON10**; 6: **2Ts-ON11**; 7: **3Ts-ON12**; 8: ladder. C) Lanes are 1: **ON1**; 2: **5’-Ts-ON8**; 3: **5’-N+ON2**; 4: **2Ts-ON11**; 5: **2N+ON5**; 6: **3Ts-ON12**; 7: **3N+ON6**; 8: **4Ts-ON13**; 9: **4N+ON7**. Ladder is a mixture of polythimidylates as indicated on the gel picture.

2. ESIMS analysis of the modified ONs

ESIMS spectra were recorded using a Thermo Scientific Q-Exactive Focus Hybrid Quadrupole-Orbitrap Mass Spectrometer. The modified ONs were prepared into 4 µM strand concentration with water/methanol 4:1 (HPLC grade from Fisher Scientific). Samples (5 µL) were injected via a Dionex Ultimate 3000 HPLC system running at 0.1 mL/min CH_3OH.

A)

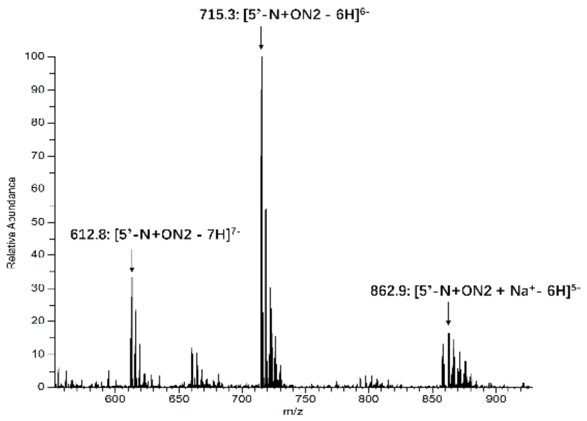

B)

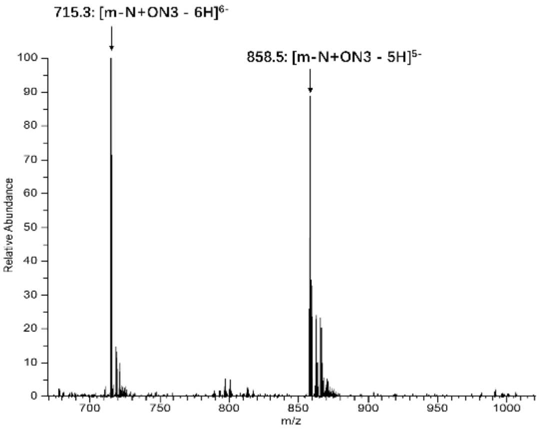

C)

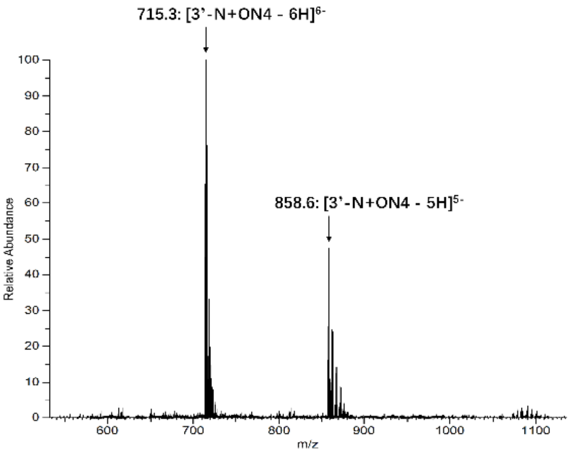

D)

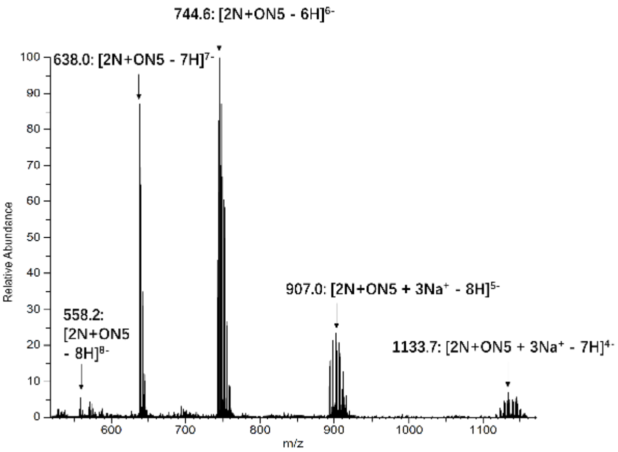

E)

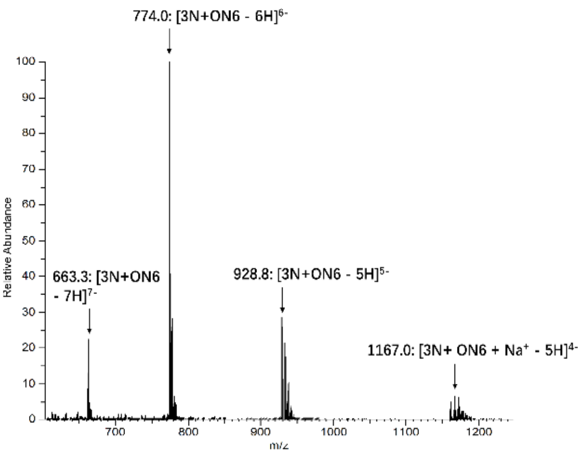

F)

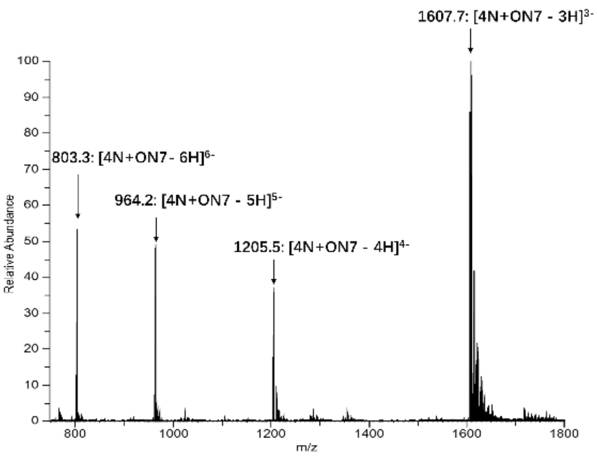

Figure S2: ESIMS results for **ON2**–**ON7**. Calculated and found molecular weights of each modified ONs are listed in Table 1.

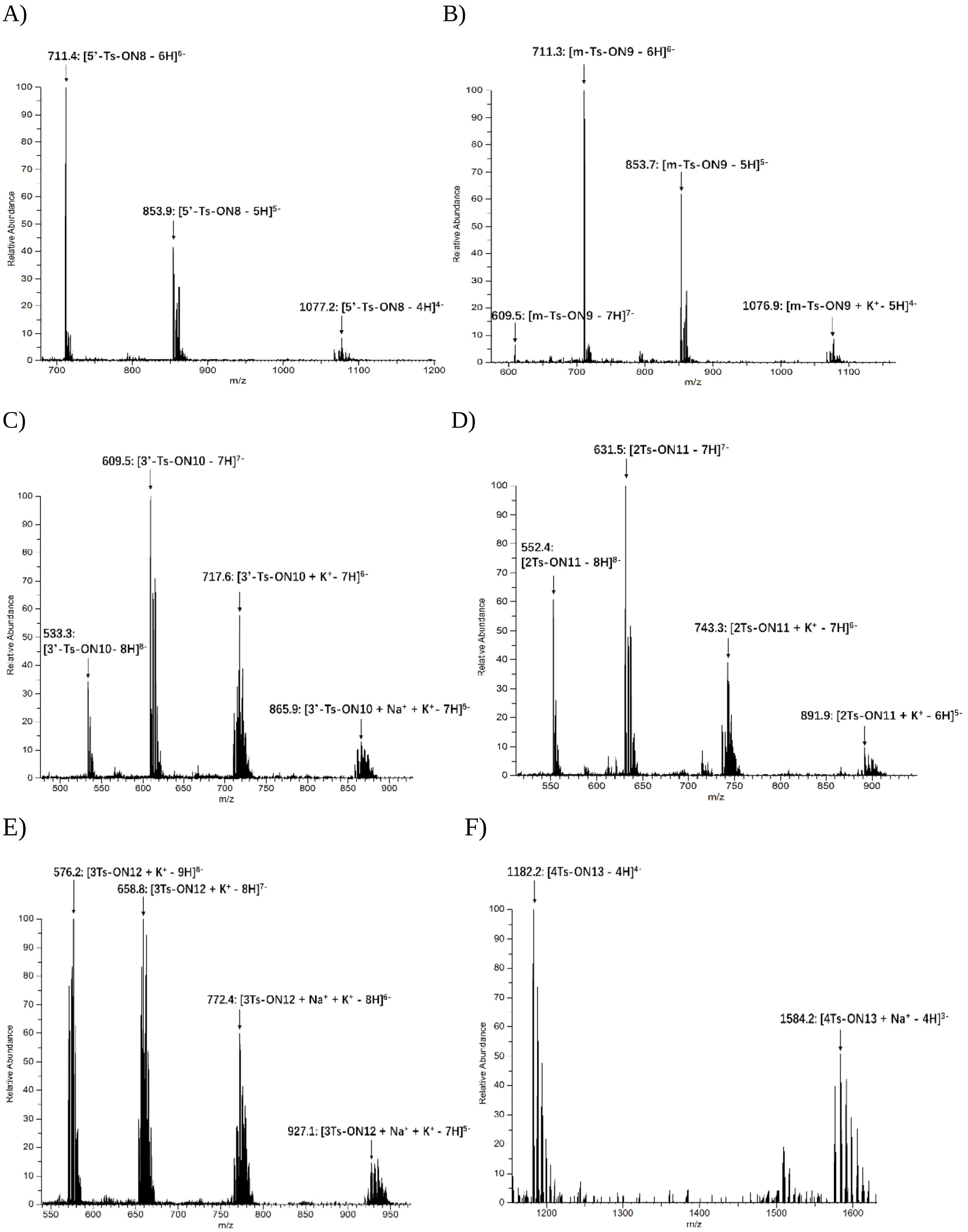

Figure S3: ESIMS results for **ON8–ON13**. Calculated and found molecular weights of each modified ONs are listed in Table 1.

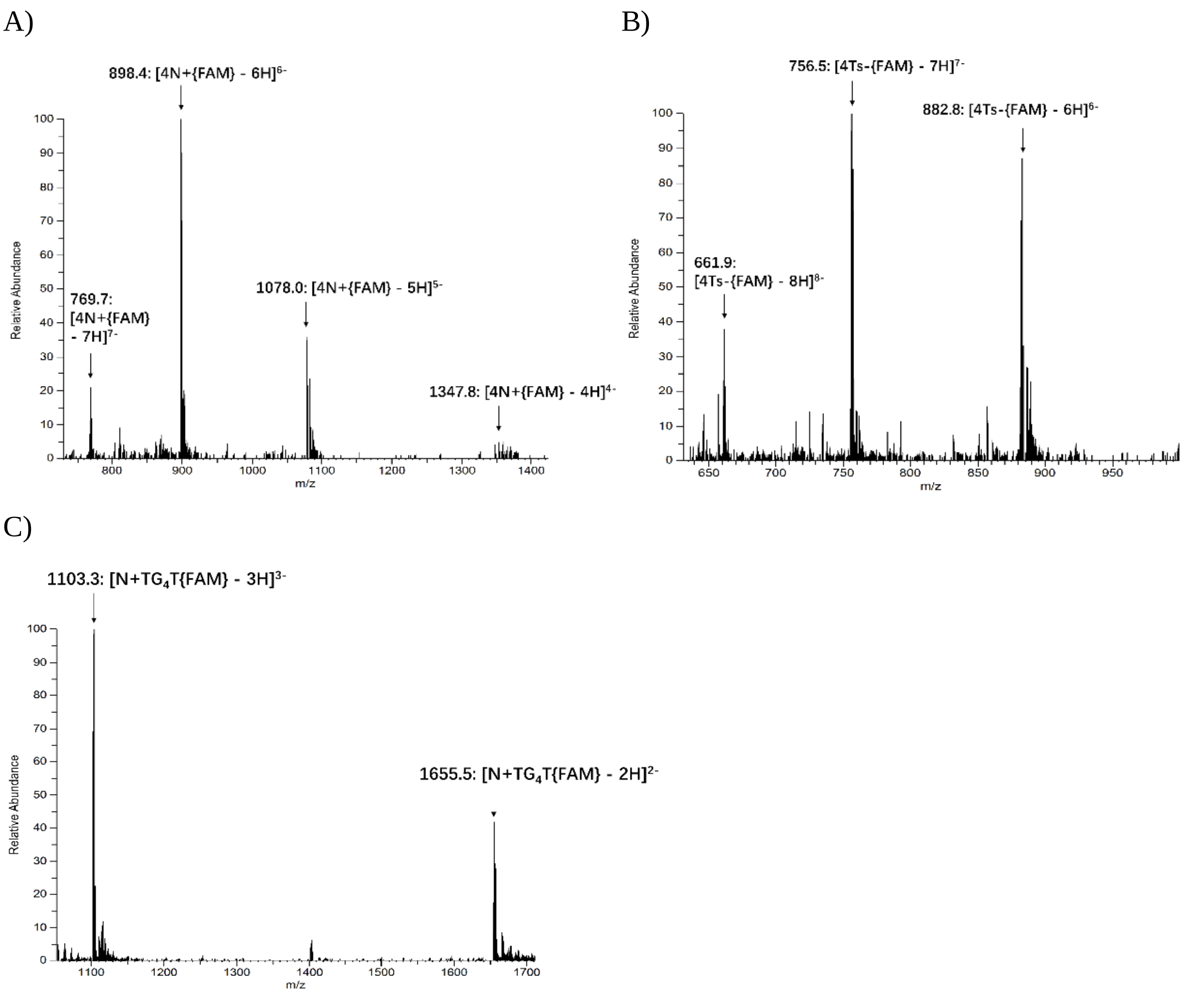

Figure S4: ESIMS results for fluorescently labelled ONs. Calculated and found molecular weights of 4N+{FAM} and 4Ts-{FAM} ONs are listed in Table 1; calculated molecular weight of $[N+TG_4T\{FAM\}\ \text{-}3H]^{3-}$ is 1103.5, observed: 1103.3.

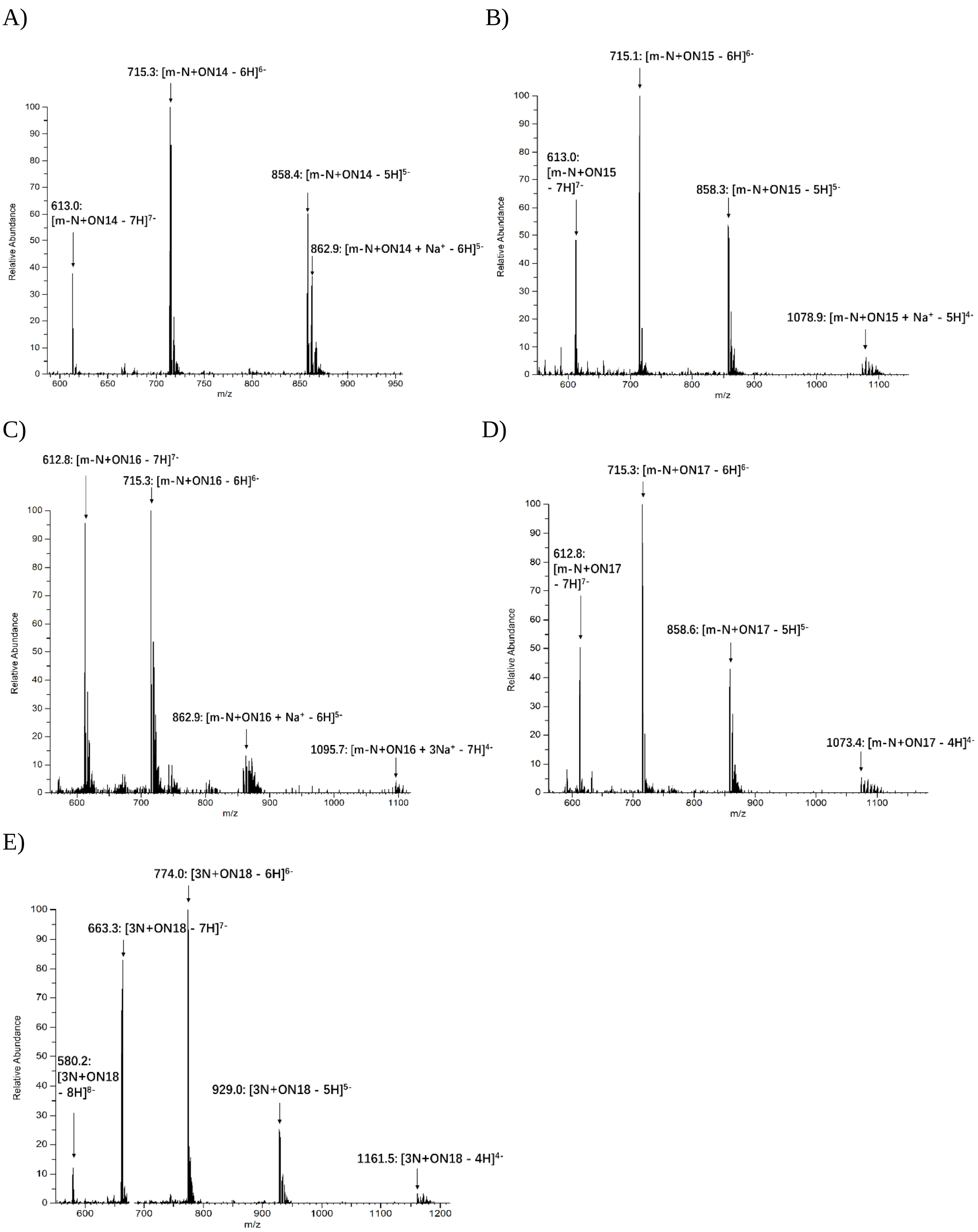

Figure S5: ESIMS results for **ON14**–**ON18**. Calculated and found molecular weights of each modified ONs are listed in Table 1.

3. Determination of the melting temperature of duplexes and triplexes using UV–vis spectrometry

Parallel and antiparallel duplexes were formed by mixing two strands (each at a concentration of 1.0 µM) in the corresponding buffer solution as listed in Table 1. Each solution was heated to 80 °C for 5 min and cooled to room temperature. Triplexes were formed by first mixing the two strands of the Watson–Crick duplex, each at a concentration of 1.0 µM in the corresponding buffer solution. The solution was heated to 80 °C for 5 min and cooled to room temperature, and the third strand (TFO) was added and then kept at 15 °C for at least 30 min.

Melting temperature measurements were performed on a Cary 100Bio UV–vis spectrometer using quartz cuvettes with 10 mm pathlengths and a 2 × 6 multicell block with a Peltier temperature controller. The melting temperature (T_m, °C) for parallel and antiparallel duplexes was determined as the maximum of the first derivative plots of the melting curves obtained by measuring the absorbance at 260 nm against increasing temperature (0.5 °C per min). The T_m values for parallel ONs/DNA duplexes is presented in Table S1.

For parallel triplex at pH 5.0 and pH 6.0, a melting curve with two transition states (Figure S6C, blue line) was obtained due to the triplex and duplex melting curves were overlaid. A melting triplex profile was obtained by subtracting the melting curve of duplex **D1** (Figure S6C, black line) from the triplex melting curve. The T_m value for a parallel triplex was determined as a cross point of the obtained triplex melting curve (Figure S6C, red line) with its median of the upper and lower baselines (Figure S6C). All melting temperatures are within the uncertainty of ± 0.5 °C as determined by repetitive experiments.

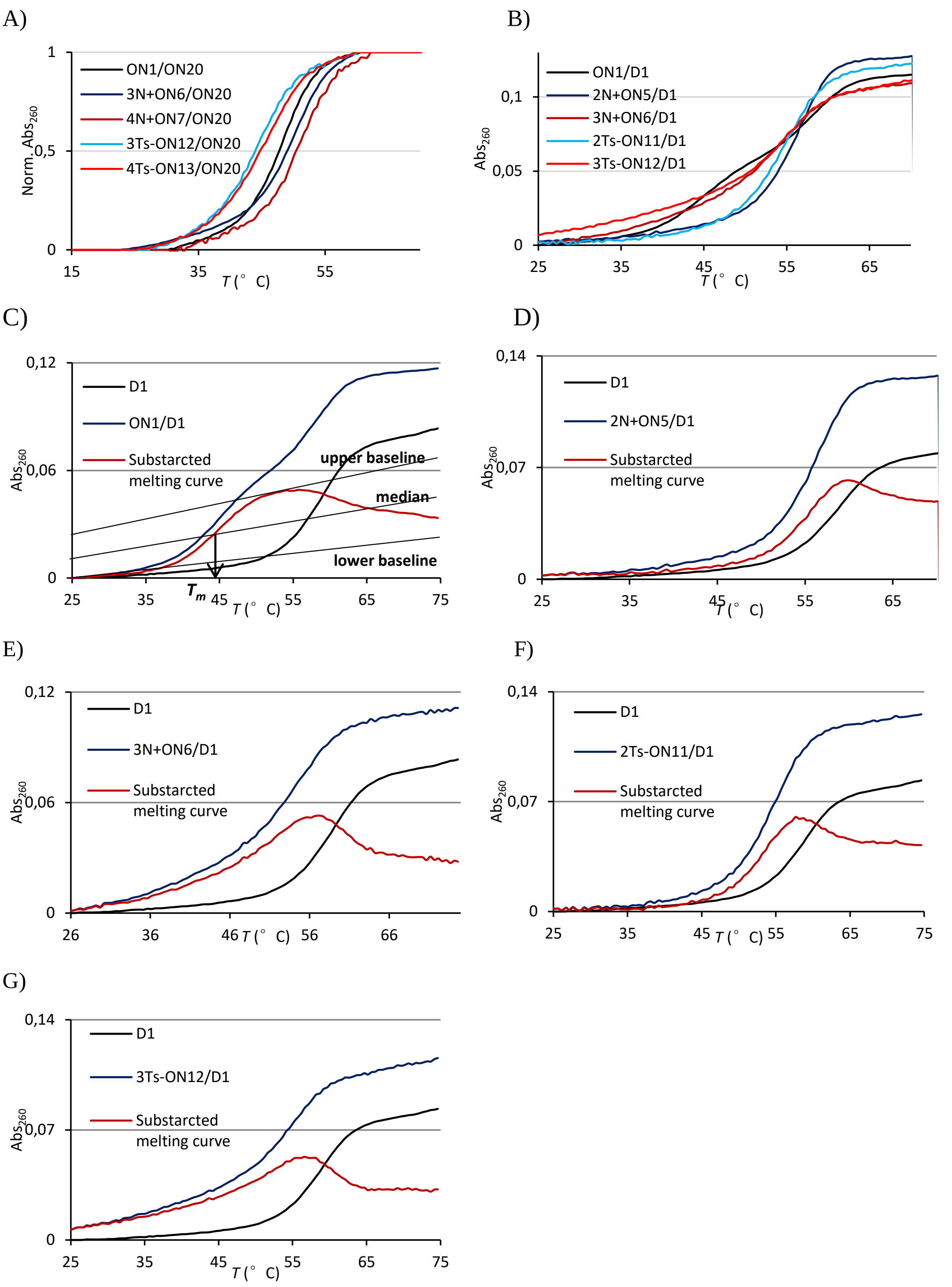

Figure S6: Representative UV–vis melting curves recorded at 260 nm against increasing temperature for A) antiparallel DNA duplexes at pH 7.0; and B) parallel triplexes at pH 5.0; C) A triplex melting profile (red solid line) obtained after subtraction of a duplex melting curve (**D1**, black line) from the triplex melting curve (**ON1:D1**, blue line). D)–G) Subtracted melting curve for parallel triplexes shown in B). Note that DNA triplex formation for **3Ts-ON12/D1** at pH 5.0 was confirmed by SE-HPLC (Figure S15).

Table S1. T_m [°C] Data for parallel duplex melting, taken from UV melting curves (λ = 260 nm).

	Sequence	Parallel duplex[a]		
		pH 5.0	pH 6.0	pH 7.0
**ON1**	5'-CCCCTTTCTTTTTT [c]	24	21	< 15
**5'-N+ON2**	5'-C$_{N+}$CCCTTTCTTTTTT	32 (+ 8.0)	< 15	< 15
**m-N+ON3**	5'-CCCCTTT$_{N+}$CTTTTTT	33 (+ 9.0)	20 (- 1.0)	20
**3'-N+ON4**	5'-CCCCTTTCTTTTT$_{N+}$T	33 (+ 9.0)	20 (- 1.0)	21
**2N+ON5**	5'-C$_{N+}$CCCTTTCTTTTT$_{N+}$T	32 (+ 8.0)	21 (0.0)	20
**3N+ON6**	5'-C$_{N+}$CCCTTT$_{N+}$CTTTTT$_{N+}$T	32 (+ 8.0)	22 (+ 1.0)	21
**4N+ON7**	5'-C$_{N+}$CCCT$_{N+}$TTCT$_{N+}$TTTT$_{N+}$T	31 (+ 7.0)	19 (- 2.0)	19
**5'-Ts-ON8**	5'-C$_{Ts}$CCCTTTCTTTTTT	39 (+ 15.0)	< 15	< 15
**m-Ts-ON9**	5'-CCCCTTT$_{Ts}$CTTTTTT	16 (- 8.0)	17 (- 4.0)	< 15
**3'-Ts-ON10**	5'-CCCCTTTCTTTTT$_{Ts}$T	32 (+ 8.0)	19 (- 4.0)	20
**2Ts-ON11**	5'-C$_{Ts}$CCCTTTCTTTTT$_{Ts}$T	30 (+ 6.0)	19 (- 4.0)	18
**3Ts-ON12**	5'-C$_{Ts}$CCCTTT$_{Ts}$CTTTTT$_{Ts}$T	18 (- 6.0)	21 (0.0)	< 15
**4Ts-ON13**	5'-C$_{Ts}$CCCT$_{Ts}$TTCT$_{Ts}$TTTT$_{Ts}$T	16 (- 8.0)	24 (+ 3.0)	< 15

[a] DNA sequence for parallel duplex formation is 5'-GGGGAAAGAAAAAA; C = 1.0 μM of each strand in 20 mM sodium cacodylate, 100 mM NaCl, 10 mM $MgCl_2$, pH 5.0, 6.0 or 7.0.

3.1. Determination of melting temperature using CD measurements

To confirm the T_m values of antiparallel duplexes formed by ONs/RNA obtained from UV–vis denaturation experiment, CD denaturation experiments were conducted using the same buffer and DNA concentrations as for the UV–vis measurements. The CD spectra were recorded using a Chirascan CD spectrophotometer (150 W Xe arc) from Applied Photophysics with a Quantum Northwest TC125 temperature controller. The CD spectra (average of at least 3 scans) were recorded between 220 and 350 nm with 1 nm intervals, 120 nm/min scan rate and 10 mm path length followed by subtraction of a background spectrum (buffer only). CD denaturation and renaturation experiments were performed by recording spectra every 2.5 °C with equilibration for 2.5 min at each temperature from 10 to 75 °C (Figure S7A).

The comparison of T_m values obtained from UV–vis and CD melting experiments is shown in Table S2.

A) B)

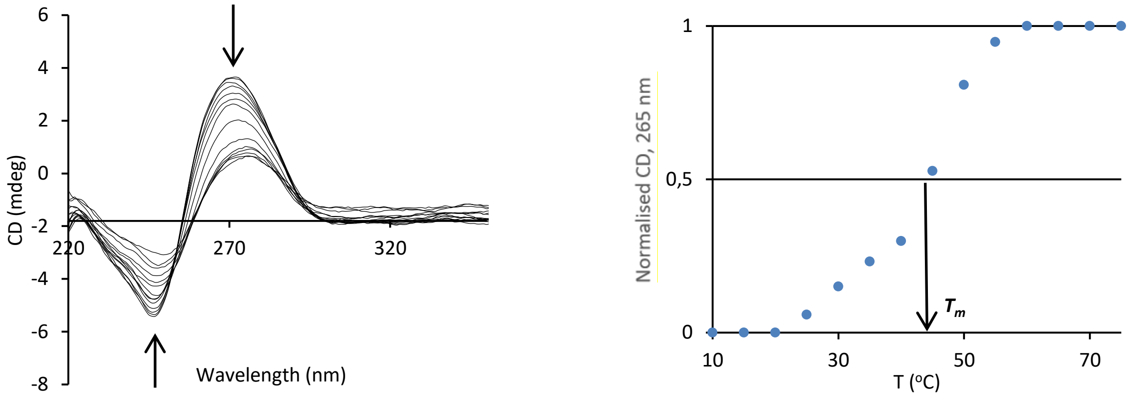

Figure S7: A) CD melting profile of DNA/RNA duplex **ON1**:**ON16** from 10–75 °C; the arrow indicates the direction of changes in the peak increasing from low to high temperatures. B) Plotted melting curve of extracted data at 265 nm against temperature.

A) B)

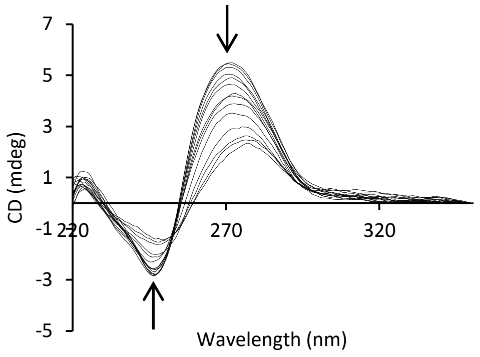

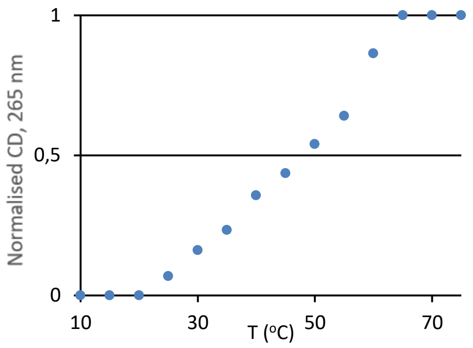

C)

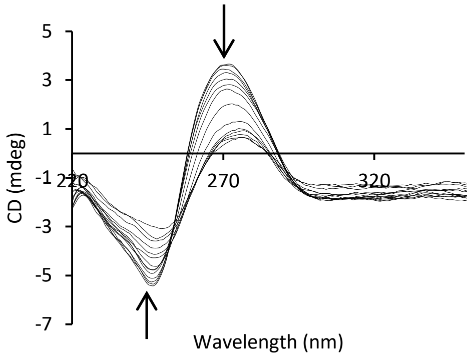

D)

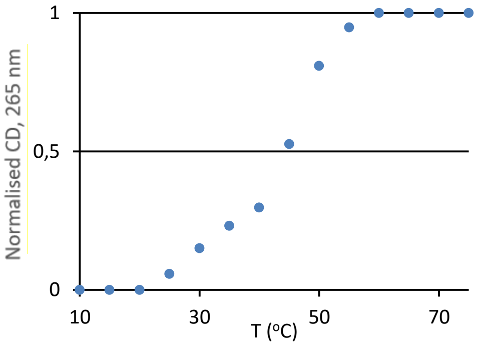

E)

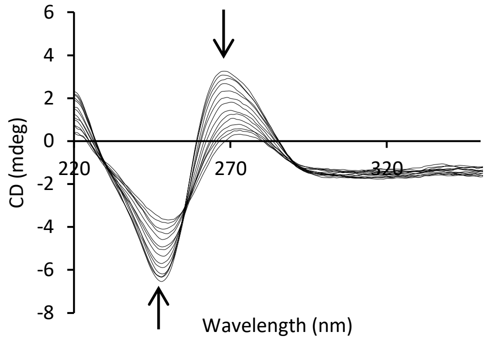

F)

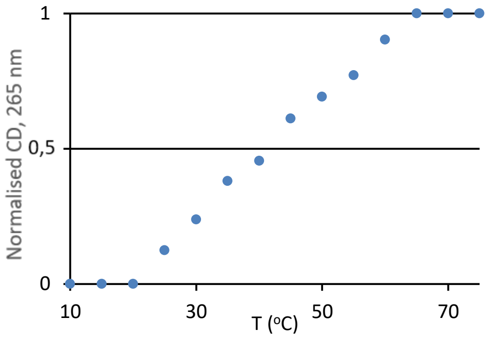

G)

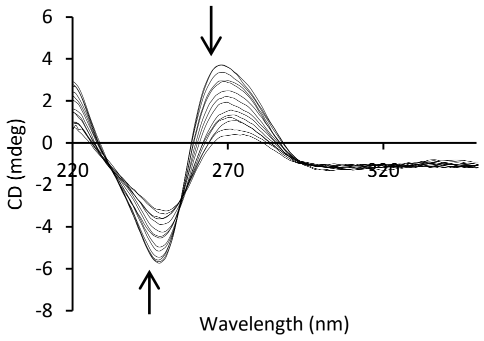

H)

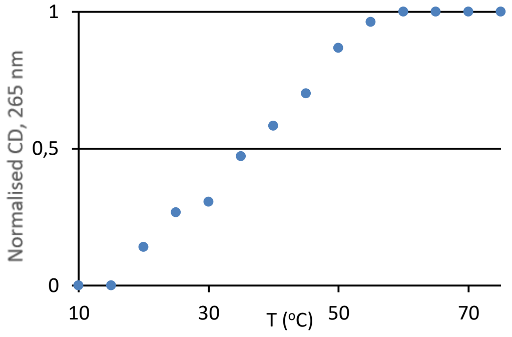

Figure S8: Left: CD melting profiles of DNA/RNA duplexes, A) **m-N+ON3/ON19**; C) **3N+ON6/ON19**; E) **m-Ts-ON9/ON19**; G) **3Ts-ON12/ON19** from 10–75 °C. The arrow indicates the direction of changes in the peak increasing from low to high temperatures. Right: Plotted melting curve of extracted data at 265 nm against temperature, B) **m-N+ON3/ON19**; D) **3N+ON6/ON19**; F) **m-Ts-ON9/ON19**; H) **3Ts-ON12/ON19**.

Table S2: Comparison of the T_m values obtained by UV and CD denaturation experiments.

	**ON19:** 3'-GCGGAAAGAAAAAA	
	UV-melting	CD-melting
**ON1:** 5'-CCCCTTTCTTTTTT	46	47
**m-N+ON3:** 5'-CCCCTTT$_{N+}$CTTTTTT	47	48
**3N+ON6:** 5'-C$_{N+}$CCCTTT$_{N+}$CTTTTT$_{N+}$T	41	41
**m-Ts-ON9:** 5'-CCCCTTT$_{Ts}$CTTTTTT	44	44
**3Ts-ON12:** 5'-C$_{Ts}$CCCTTT$_{Ts}$CTTTTT$_{Ts}$T	38	37

3.2. Determination of thermodynamic parameters of antiparallel duplexes formed at different salt concentrations

In order to analyse thermodynamic parameters of N^+ and Ts-modified ONs toward complementary DNA and RNA at different salt concentrations, melting profiles obtained from UV melting experiment were converted into a fraction folded (ϴ) vs temperature representation (Figure S9A).
The signal change at 260 nm was extracted, converted to fraction folded ϴ and plotted against temperature to give the T_m value (Figure S7B). The conversion of UV signal at 260 nm (C_T) to Θ_T at a given temperature was followed by adequate upper and lower baselines chosen:

$$\Theta_T = (L0_T - C_T) / (L0_T - L1_T) \quad \text{(Equation 1)}$$

$L0_T$ and $L1_T$ correspond to the baseline values of the unfolded and folded species, respectively. ϴ is a number between 0 and 1: ϴ = 0 for T>>T_m, ϴ = 1 for T<<T_m, and ϴ = 0.5 for T = T_m.
By definition, the free Gibbs enthalpy may be written as:

$$\triangle G^0 = -RT \ln(K_a) = \triangle H^0 - T \times \triangle S^0 \quad \text{(Equation 2)}$$

Where R = 8.3145 J/(K·mol), T is the temperature in Kelvin, $\triangle H^0$ is the standard enthalpy of the reaction, and $\triangle S^0$ is the standard entropy, assuming that $\triangle c_p = 0$[4]
Equation 2 can be deduced as:

$$\ln(K_a) = -\triangle H^0 / R \times (1/T) + \triangle S^0 / R \quad \text{(Equation 3)}$$

Therefore, the following step required a van't Hoff plot of the natural logarithm of the affinity constant ($\ln(K_a)$) as a function of the reciprocal of the temperature ($1/T$ in K^{-1})[5].
For bimolecular equilibrium A + B ⇔ C:

$$K_a = [C] / [A] [B]$$

$$= (C_c \times \Theta) / ([C_A \times (1 - \Theta)] \times [C_B \times (1 - \Theta)]) \qquad \text{(Equation 4)}$$

When A and B is present at the same initial strand concentration C_0

$$K_a = \Theta / (C_0 \times (1 - \Theta)^2) \qquad \text{(Equation 5)}$$

where C_0 is the initial strand concentration and Θ is Θ_T at each temperature. It should be noted that the analysis should be restricted between the temperature range for which $0.03 < \Theta < 0.97$ as it is relatively difficult to evaluate the affinity constant when almost all or almost none of the molecules are associated[6].

Following the calculations described above, Figure S9A was converted into Figure S9B. The van't Hoff relation (ln(K_a) vs. 1/T) should give a straight line, with a slope of $-\triangle H^0/R$ and the y-axis intercept of $\triangle S^0/R$. The same procedure was used for determination of thermodynamic parameters for all complexes studied in Table 3.

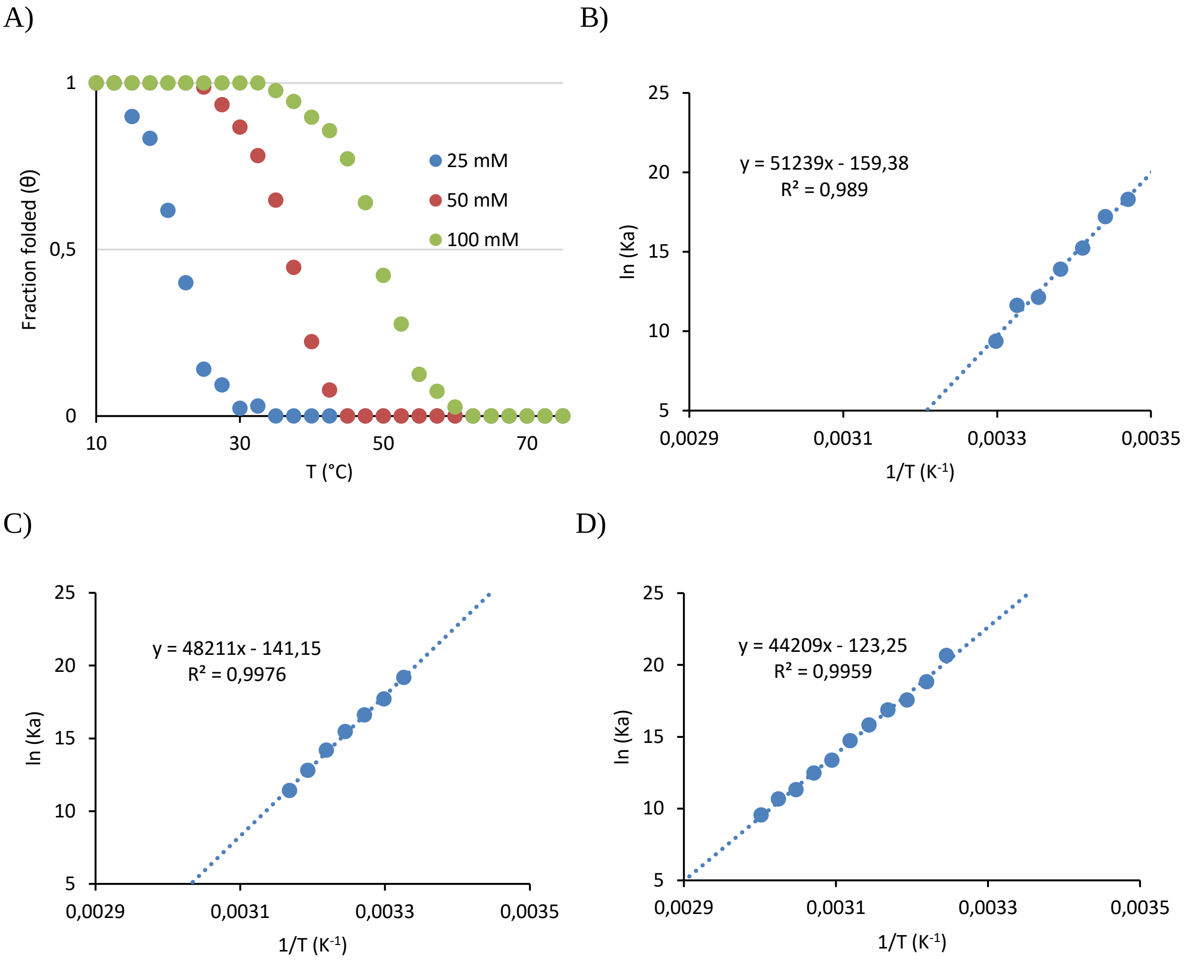

Figure S9: Determination of thermodynamic parameters. A) Fraction folded as a function of temperature for control **ON1/ON20** duplex. B), C) and D) van't Hoff plot used for determination of $\triangle H^0$ and $\triangle S^0$ at 25, 50 and 100 mM NaCl concentration used respectively. Θ values are extracted from the melting curve obtained by UV–vis denaturation experiments. Not all Θ points are plotted in this figure, only Θ values significantly higher than 0 and lower than 1 are used.

A)

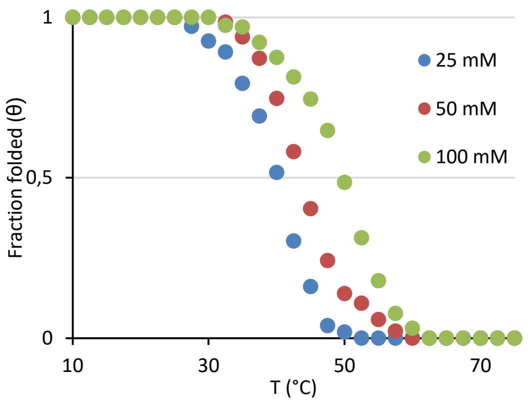

B)

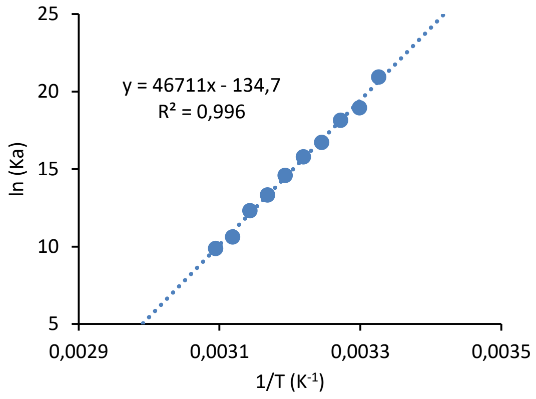

C)

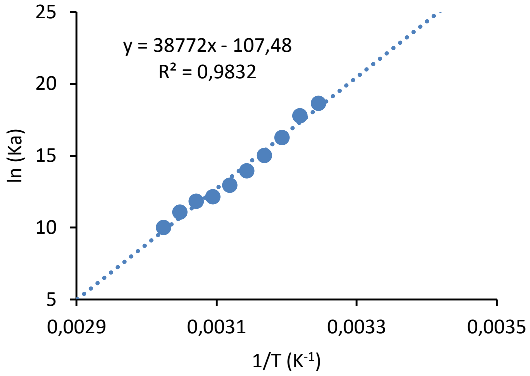

D)

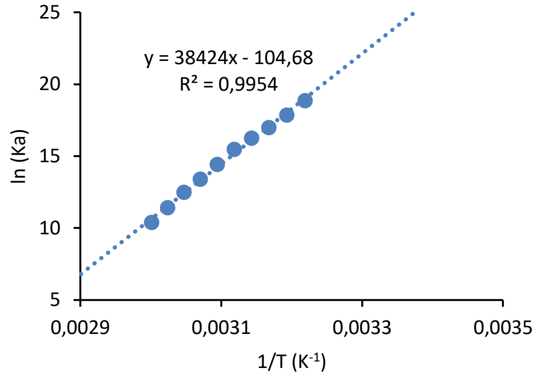

Figure S10: Determination of thermodynamic parameters. A) Fraction folded as a function of temperature for **4N+ON7/ON20** duplex. B), C) and D) van't Hoff plot used for determination of $\triangle H^0$ and $\triangle S^0$ at 25, 50 and 100 mM NaCl concentration used, respectively. Θ values are extracted from the melting curve obtained by UV–vis denaturation experiments. Not all Θ points are plotted in this figure, only Θ values significantly higher than 0 and lower than 1 are used.

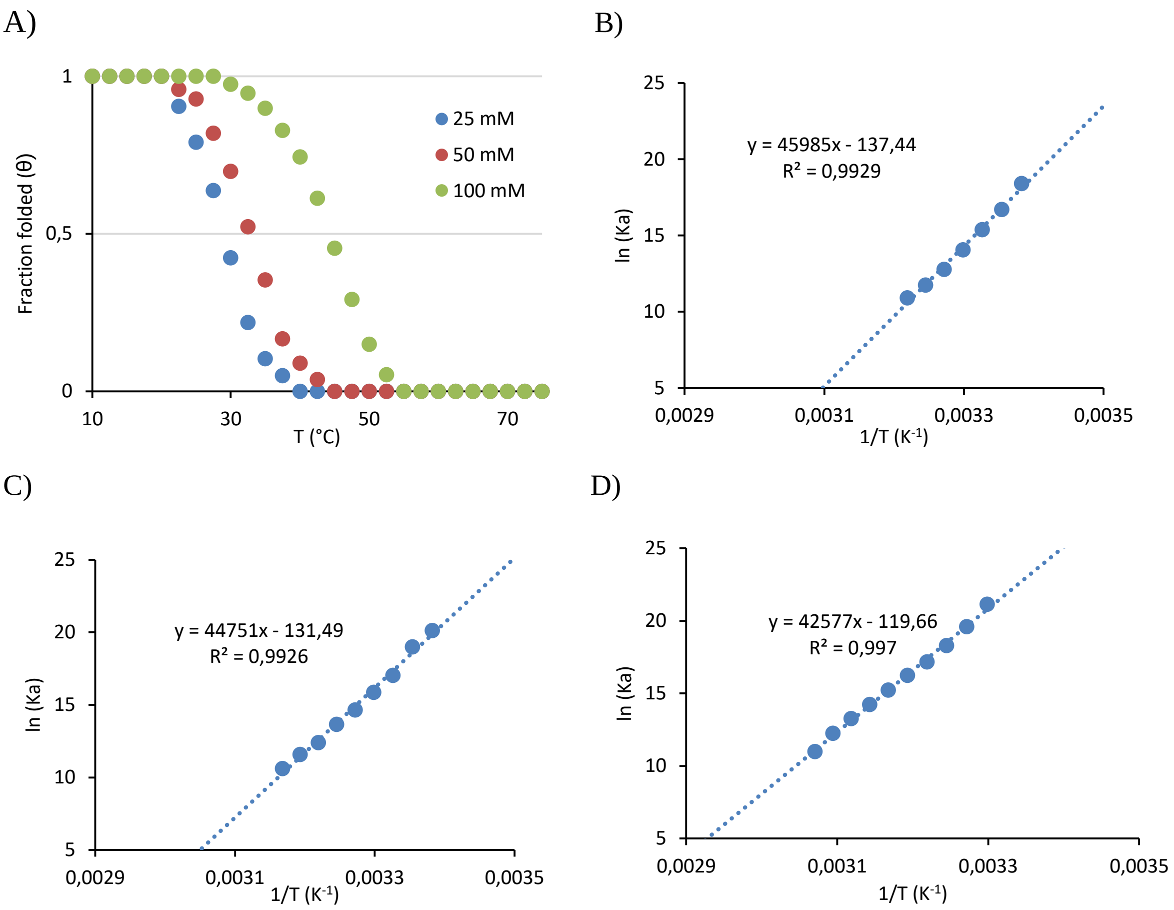

Figure S11: Determination of thermodynamic parameters. A) Fraction folded as a function of temperature for **4Ts-ON13/ON20** duplex. B), C) and D) van't Hoff plot used for determination of $\triangle H^0$ and $\triangle S^0$ at 25, 50 and 100 mM NaCl concentration used, respectively. Θ values are extracted from the melting curve obtained by UV–vis denaturation experiments. Not all Θ points are plotted in this figure, only Θ values significantly higher than 0 and lower than 1 are used.

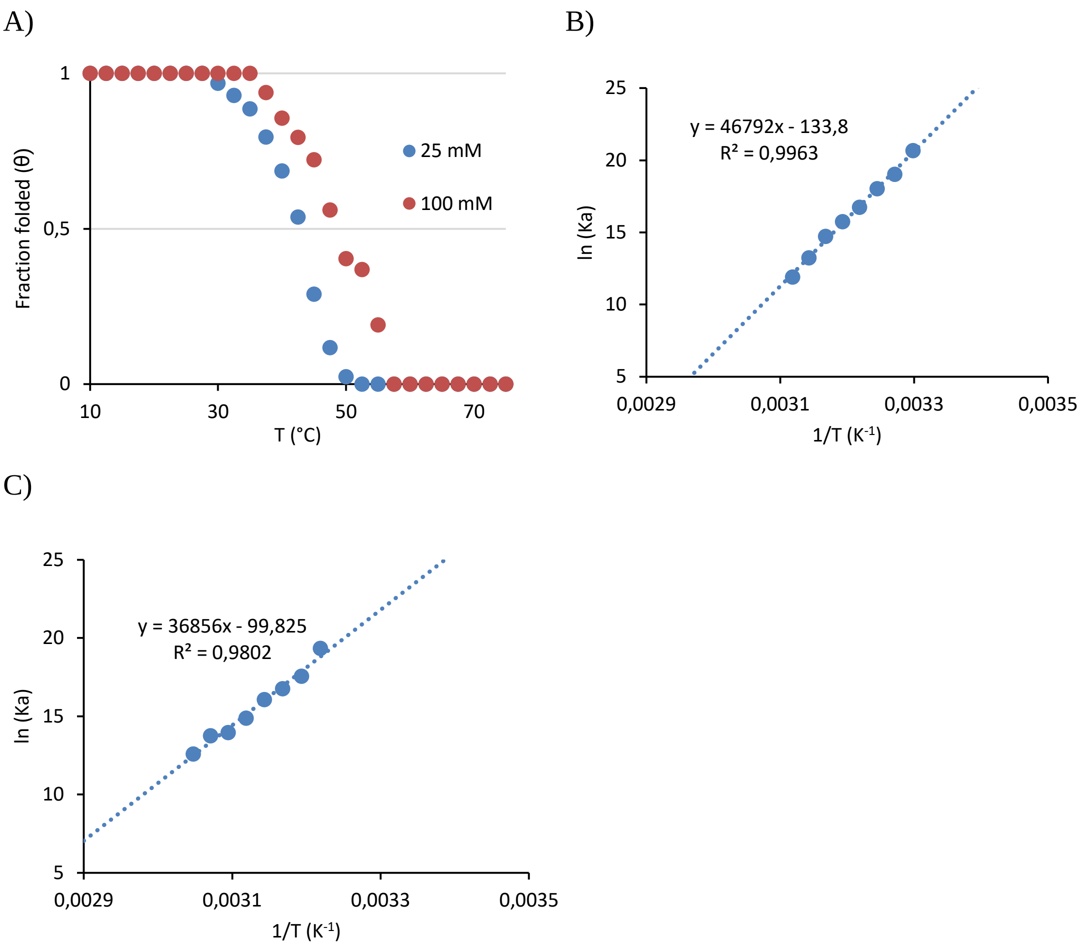

Figure S12: Determination of thermodynamic parameters. A) Fraction folded as a function of temperature for control ON/RNA duplex: **ON1/ON19**. B), C) and D) van't Hoff plot used for determination of $\triangle H^0$ and $\triangle S^0$ at 25, 50 and 100 mM NaCl concentration used respectively. ϴ values are extracted from the melting curve obtained by UV-Vis denaturation experiments. Not all ϴ points are plotted in this figure, only ϴ values significantly higher than 0 and lower than 1 are used.

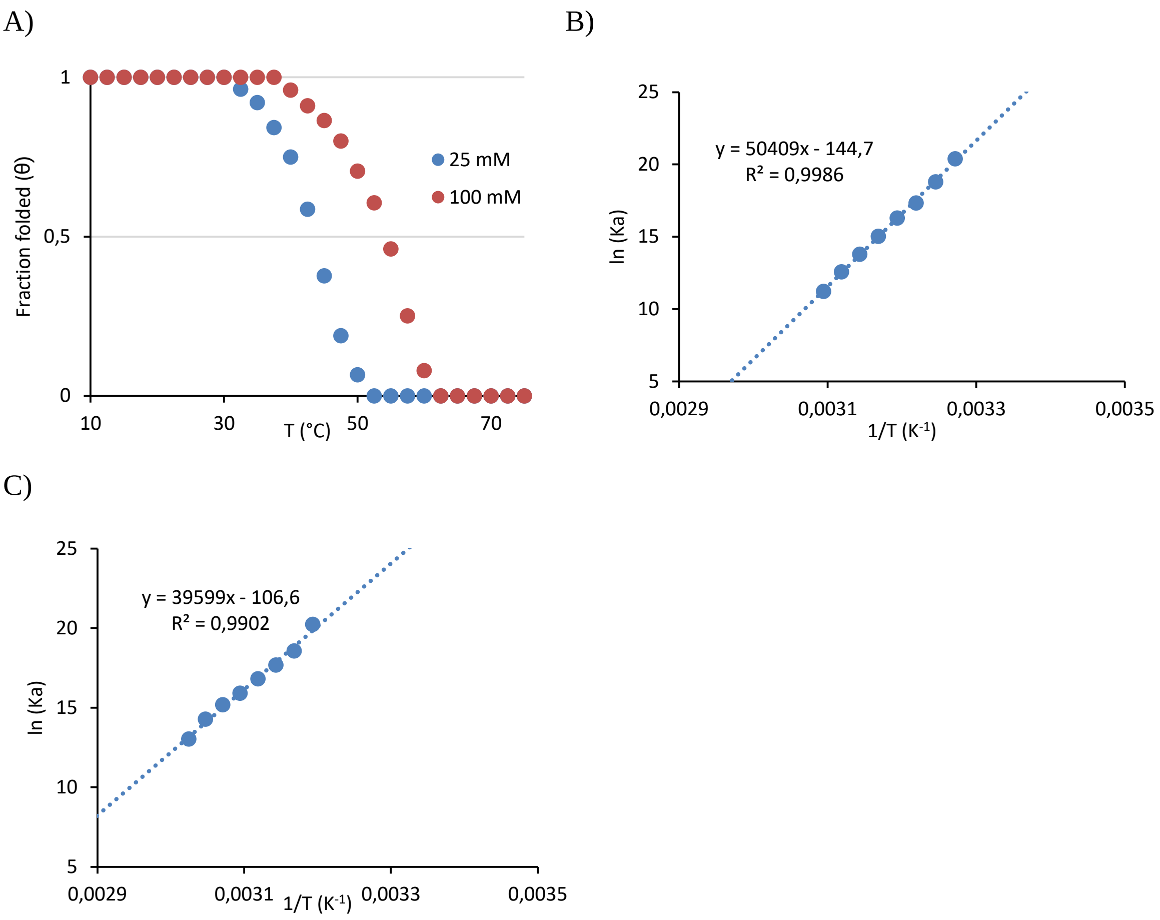

Figure S13: Determination of thermodynamic parameters. A) Fraction folded as a function of temperature for **4N+ON7/ON19** duplex. B), C) and D) van't Hoff plot used for determination of $\triangle H^0$ and $\triangle S^0$ at 25, 50 and 100 mM NaCl concentration used, respectively. Θ values are extracted from the melting curve obtained by UV–vis denaturation experiments. Not all Θ points are plotted in this figure, only Θ values significantly higher than 0 and lower than 1 are used.

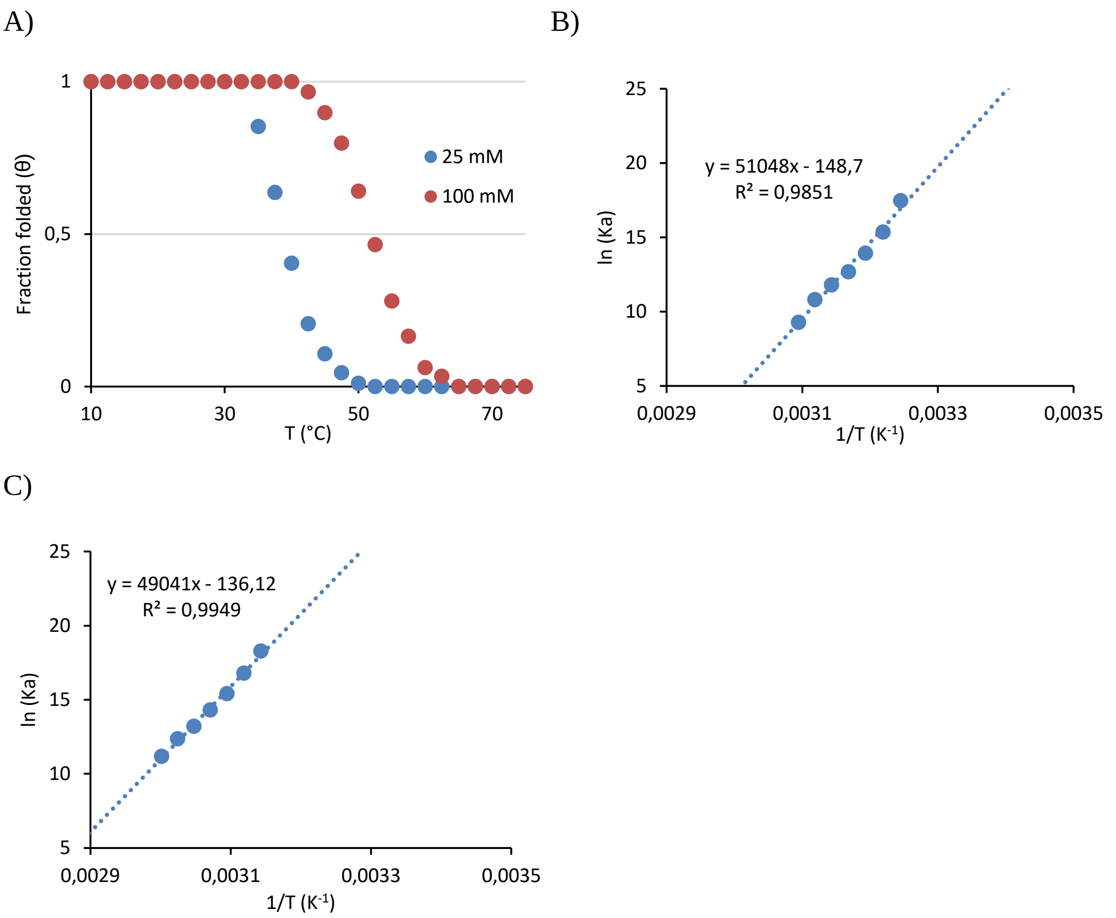

Figure S14: Determination of thermodynamic parameters. A) Fraction folded as a function of temperature for **4Ts-ON13/ON19** duplex. B), C) and D) van't Hoff plot used for determination of $\triangle H^0$ and $\triangle S^0$ at 25, 50 and 100 mM NaCl concentration used respectively. Θ values are extracted from the melting curve obtained by UV–vis denaturation experiments. Not all Θ points are plotted in this figure, only Θ values significantly higher than 0 and lower than 1 are used.

4. Evaluation of triplex formation using size-exclusion HPLC

To confirm triplex formation, we performed size-exclusion (SE) HPLC evaluation of several samples at pH 50 and 6.0 at rt (25 °C). Preformed complex samples (1 µM, 100 µL) were analysed on an Ultimate 3000 HPLC system, equipped with an autosampler, a diode array detector detecting absorbance at 260 nm and a Thermo Acclaim SEC-300 column (4.6 × 300 mm; 5-µm hydrophilic polymethacrylate resin spherical particles, 300 Å pore size). 10 mM Na-cacodylate buffer (pH 5.0 and pH 6.0, respectively) supplemented with 100 mM NaCl and 10 mM $MgCl_2$ was used as a mobile phase.

A) B)

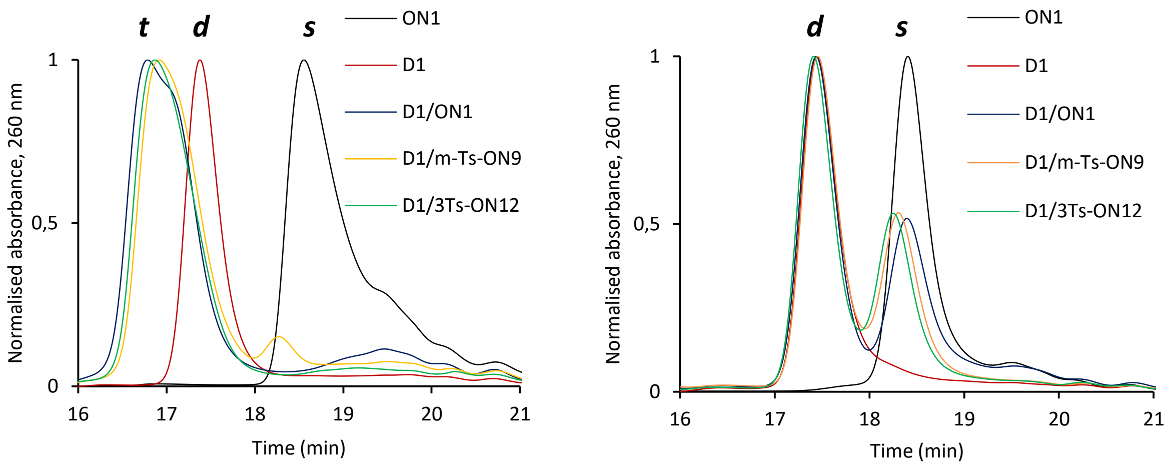

Figure S15. A) SE-HPLC profiles confirming formation of a triplex at pH 5.0; B) SE-HPLC spectra confirming no triplex formation at pH 6.0. Triplexes are indicated with a *t*, duplexes with a *d*, and *s* stands for single-stranded DNA.

As shown in Figure S15B, samples of **D1/ON1**, **D1/m-Ts-ON9** and **D1/3Ts-ON12** at pH 6.0 showed no triplex formation at rt (25 °C). Only peaks corresponding to the duplex **D1** (17.4 min) and single-stranded DNAs (ssDNAs, ≈ 18.5 min) were observed. ssDNAs have similar retention time to **ON1** (18.3 min) in SE-HPLC. In contrast, the same samples at pH 5.0 showed new peaks with faster retention time at 16.8 min corresponding to DNA triplexes.

5. Evaluation of the stability of 5’N+ON2 in Na-phosphate buffer at various pH at 55 °C

5’N+ON2 (1.0 µM) was incubated at 55 ºC in 20 mM Na-phosphate buffer (100 mM NaCl, 10 mM $MgCl_2$, pH 5.5, 7.0 and 8.5, respectively) for 24 h. Samples were analysed using IE-column (TSKgel Super Q-5PW). Buffer A [20 mM Tris·HCl, 1 mM Na_2-EDTA, pH 9.0], buffer B [20 mM Tris·HCl, 1 mM Na_2-EDTA, 1M NaCl, pH 9.0]. Gradients: 3.7 min 100% A, convex curve gradient to 30% B in 11.1 min, linear gradient to 50% B in 18.5 min, concave gradient to 100% B in 7.4 min, keep 100% B for 7.4 min and then 100% A in 7.3 min.

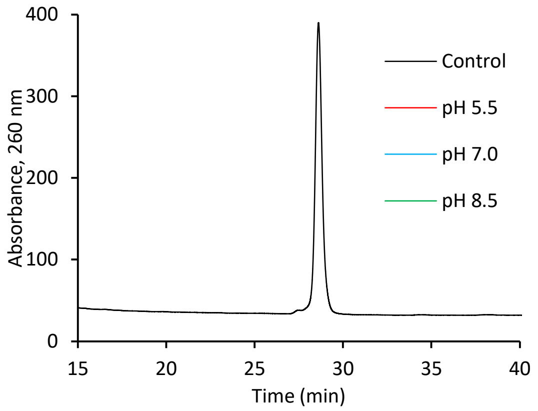

Figure S16: IE-HPLC profiles of samples of **5’-N+ON2** after incubation at 55 ºC in Na-phosphate buffer (pH 5.5, 7.0 and 8.5, respectively) for 24 h. Control is a sample of **5’-N+ON2** in Na-phosphate buffer (pH 7.0) without incubation at 55 ºC.

6. Enzymatic digestion of ONs by phosphodiesterase I

The nuclease stability of the modified ONs was evaluated using snake venom phosphodiesterase (phosphodiesterase I, Sigma, made into 0.8 units /mL stock) and compared with unmodified sequence **ON1**. N+ONs, Ts-ONs and **ON1** at 7.5 µM were incubated with phosphodiesterase I (0.16 units /mL, 12 µL) in 60 µL of 5 mM Tris·HCl buffer (pH 8.0, 10 mM $MgCl_2$) at 37 °C. 10 µL aliquots were collected at 0, 10, 30, 60 and 120 min, heated at 90 °C for 5 min to deactivate the enzyme and analysed by SE-HPLC to evaluate the amount of intact ONs remaining (Figure S17). The percentage of intact ONs in each sample was calculated and plotted against the digestion time (Fig. 2 in the main text). Percentage of intact ONs was determined by the ratio of full-length ONs at each time evaluated to the sample at 0 min.

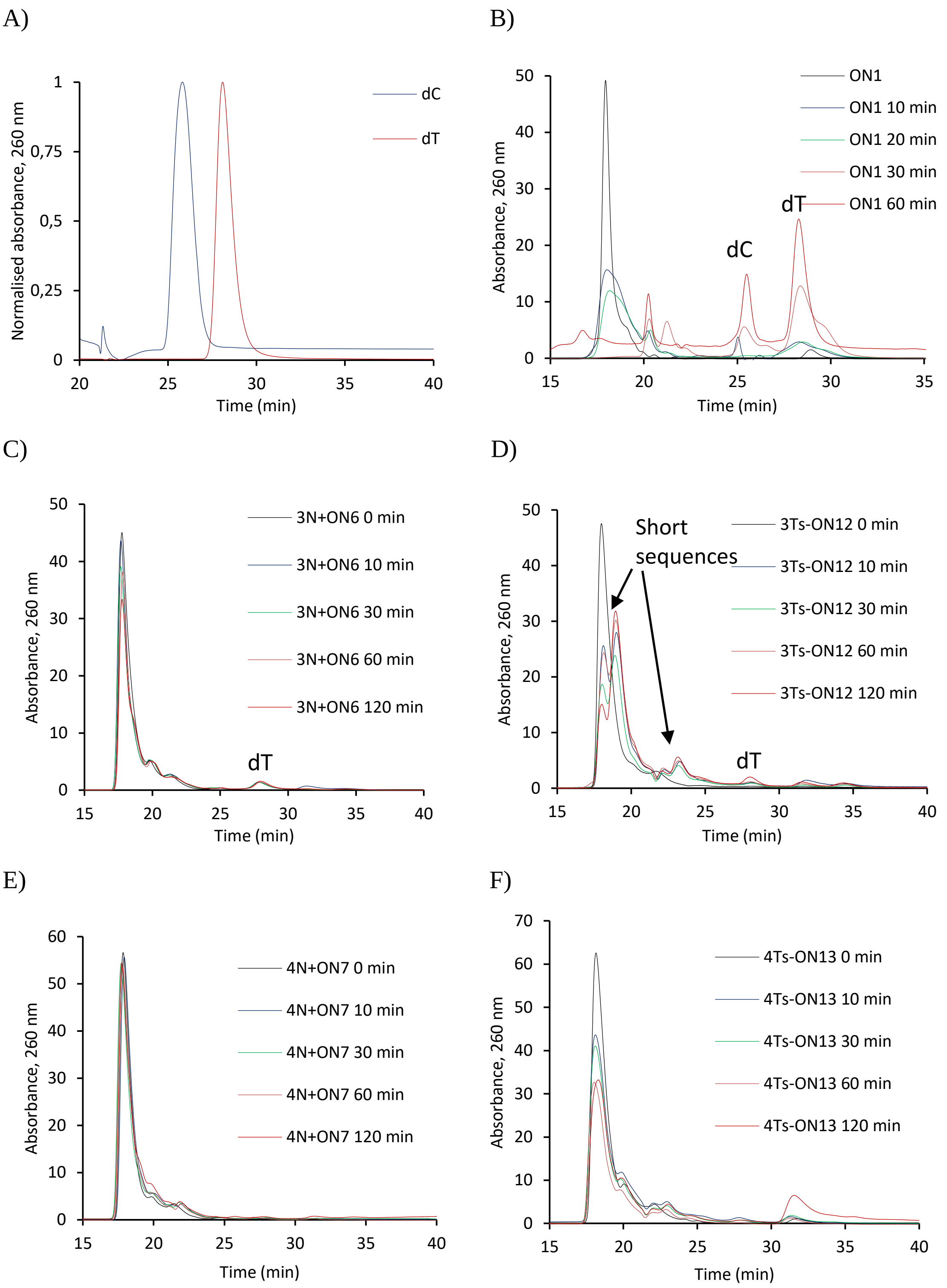

Figure S17: SE-HPLC profiles of A) 2'-deoxycytidine (dC) and thymidine (dT); B) - F) ONs after incubation in 5 mM Tris·HCl (pH 8.0, 10 mM $MgCl_2$) containing 0.16 units/mL of snake venom phosphodiesterase at 0, 10, 30, 60 and 120 min, respectively.

7. Cell culture assay

The mouse fibroblast cell line NIH 3T3 (ATCC) was maintained in DMEM (Gibco, Thermofisher Scientific) with 1% penicillin/streptomycin (Gibco) and 10% calf serum (Thermofisher Scientific) at 37 °C with 5% CO_2 in a humidified atmosphere.

Ts and N+-modified FAM-labelled ONs were diluted in Opti-MEM medium (Gibco) and then added to a final concentration of 20 µM to NIH 3T3 cells growing asynchronously on fibronectin coated coverslips. After 12 h, cells were washed with Dulbecco's phosphate buffered saline with Ca^{2+} and Mg^{2+} (DPBS, Thermofisher Scientific), fixed in 4% paraformaldehyde, washed again with DPBS before staining with 1 µg/mL Hoechst 3342. The coverslips were then rinsed in DPBS and mounted in Slowfade Diamond Antifade Mountant (Invitrogen, Thermofisher Scientific) on a microscope slide. For membrane staining, cells were stained with CellBrite Fix 640 (Biotium) for 15 min at rt before fixation and then processed as above.

Slides were imaged using a Leica SP5 DM6000B Scanning Confocal Microscope equipped with 63x/1.40 objective lens. Probes were excited with 405 nm, 496 nm and 633 nm excitation lasers, running LAS X software (Leica) and digitally processed for presentation with Affinity Designer v1.6.1 (Serif Ltd).

8. References

[1] Su, Y. D., Edwards, P. J. B., Stetsenko, D. A., and Filichev, V. V. (2020), *ChemBioChem 21*, 2455-2466.

[2] Prokhorova, D. V., Chelobanov, B. P., Burakova, E. A., Fokina, A. A., and Stetsenko, D. A. (2017), *Russ. J. Bioorg. Chem. 43*, 38-42.

[3] Hazen, G. G., Bollinger, F. W., Roberts, F. E., Russ, W. K., Seman, J. J., and Staskiewicz, S. (1996), *Organic Synthesis, Vol 73 73*, 144-151.

[4] Mergny, J. L., and Lacroix, L. (2003), *Oligonucleotides 13*, 515-537.

[5] Mills, M., Arimondo, P. B., Lacroix, L., Garestier, T., Hélène, C., Klump, H., and Mergny, J.-L. (1999), *J. Mol. Biol. 291*, 1035-1054.

[6] Puglisi, J. D., and Tinoco, I., Jr. (1989), *Methods Enzymol. 180*, 304-325.
